# Cathepsin B Processing Is Required for the *In Vivo* Efficacy of Albumin–Drug Conjugates

**DOI:** 10.1021/acs.bioconjchem.3c00478

**Published:** 2024-02-12

**Authors:** Barbara Bernardim, João Conde, Tuuli Hakala, Julie B. Becher, Mary Canzano, Aldrin V. Vasco, Tuomas P. J. Knowles, Jason Cameron, Gonçalo J. L. Bernardes

**Affiliations:** † Yusuf Hamied Department of Chemistry, University of Cambridge, Lensfield Road, CB2 1EW Cambridge, United Kingdom; ‡ Instituto de Medicina Molecular João Lobo Antunes, Faculdade de Medicina, Universidade de Lisboa, Avenida Professor Egas Moniz, 1649-028 Lisboa, Portugal; § Albumedix Ltd, Mabel Street, Nottingham NG2 3ED, United Kingdom

## Abstract

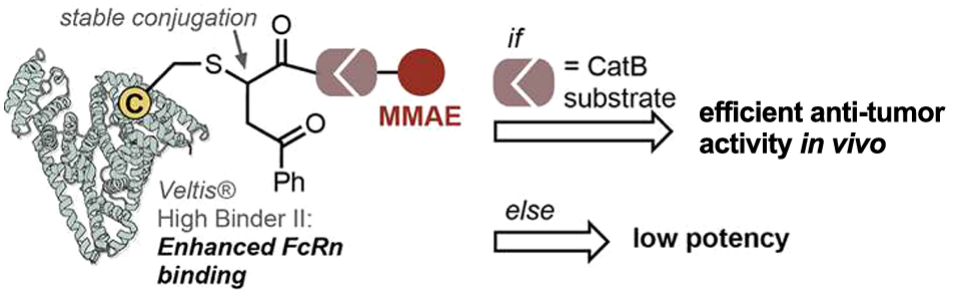

Targeted drug delivery approaches that selectively and preferentially deliver therapeutic agents to specific tissues are of great interest for safer and more effective pharmaceutical treatments. We investigated whether cathepsin B cleavage of a valine–citrulline [VC(*S*)]-containing linker is required for the release of monomethyl auristatin E (MMAE) from albumin–drug conjugates. In this study, we used an engineered version of human serum albumin, Veltis High Binder II (HBII), which has enhanced binding to the neonatal Fc (fragment crystallizable) receptor (FcRn) to improve drug release upon binding and FcRn-mediated recycling. The linker–payload was conjugated to cysteine 34 of albumin using a carbonylacrylic (caa) reagent which produced homogeneous and plasma stable conjugates that retained FcRn binding. Two caa–linker–MMAE reagents were synthesized—one with a cleavable [VC(*S*)] linker and one with a noncleavable [VC(*R*)] linker—to question whether protease-mediated cleavage is needed for MMAE release. Our findings demonstrate that cathepsin B is required to achieve efficient and selective antitumor activity. The conjugates equipped with the cleavable [VC(*S*)] linker had potent antitumor activity *in vivo* facilitated by the release of free MMAE upon FcRn binding and internalization. In addition to the pronounced antitumor activity of the albumin conjugates *in vivo*, we also demonstrated their preferable tumor biodistribution and biocompatibility with no associated toxicity or side effects. These results suggest that the use of engineered albumins with high FcRn binding combined with protease cleavable linkers is an efficient strategy to target delivery of drugs to solid tumors.

Cancer is a major cause of mortality in the European Union (EU) with more than 3.7 million new cases and 1.9 million deaths each year. This figure is anticipated to rise in the next few decades since the majority (∼60%) of people diagnosed with cancer are over 65 years in age. In fact, around one in three people in the EU will be diagnosed with cancer during their lifetime.^1^

When referring to cancer therapy, selective targeting and delivery are of utmost importance to enhance the therapeutic effect and decrease undesirable distribution to healthy organs and tissues. Nevertheless, conventional antibody–drug conjugates may suffer from premature drug release and limited efficacy.^2,3^ Therefore, the need for more effective conjugates for targeted drug delivery is imperative.

Altering the biodistribution, pharmacodynamics, and metabolism of small-molecule-based drugs through chemical modifications is routinely used to enhance their efficiency.^4^ In addition, protein-based drugs may require modification to reduce their potential immunogenicity while extending their serum half-life.^5^ More recently, for full-length immunoglobulin (IgG) antibodies^6,7^ and human serum albumin (HSA),^8,9^ enhancement of their interactions through recycling via the neonatal Fc (fragment crystallizable) receptor (FcRn) has been explored. HSA is an excellent conjugation partner because it offers both serum half-life extension (*t*_1/2_ albumin ≈ 19 days) and is recycled through the FcRn.^10^ For instance, the use of albumin for half-life extension has been demonstrated with the approvals of Eperzan, an albumin–GLP-1 fusion for the treatment of type 2 diabetes mellitus in adults,^11,12^ and IDELVION, an albumin-factor IX fusion for the treatment and prophylaxis of bleeding events and perioperative management.^13^

Because of the favorable targeting properties of albumin, various payloads have been covalently attached to this protein.^8−10^ As albumin has a single free sulfhydryl group (cysteine 34) available for conjugation, covalent modification via reaction at this position has proved to be a very popular strategy for the attachment of various payloads.^8−10^ This strategy has been used to extend the half-life of various protein-based drugs including granulocyte colony-stimulating factor (G-CSF),^14^ Kringle domain,^15^ DARPin domain,^16^ insulin,^17^ and GLP-1/exendin-4 (CJC-1131 and CJC-1134-PC).^18−20^ Lysine modification strategies have also been trialled,^8^ and despite a few examples where regioselectivity was achieved,^21−23^ these approaches often resulted in heterogeneous mixtures (due to a large number of surface accessible lysines on albumin), limited solubility (by removal of charged groups), and denatured constructs.^24^ Cysteine 34 is located near to the surface of the albumin protein in a shallow crevice. It is situated in a predominantly anionic environment and has relatively limited solvent accessibility.^25^ This environment confers some unique properties on the sulfhydryl side chain, and it has a p*K*_a_ of approximately 8.5 in the absence of external factors *in vivo*.^25^ Thus, cysteine 34 remains a residue and site of choice to achieve chemically defined albumin–drug conjugates.

The FcRn is responsible for both the long half-life of albumin and its protection from intracellular degradation through a recycling mechanism (Figure S1). It is likely that the binding potency of albumin to FcRn will interfere with recycling and determine the efficiency of drug release. Furthermore, FcRn binds albumin across species with varying affinities, which is important for design and evaluation of albumin-based therapeutics.^26^

Here, we have designed albumin–drug conjugates to provide insight into whether cathepsin B cleavage is required for release of an antineoplastic drug—monomethyl auristatin E (MMAE)—upon FcRn binding and internalization. The drug payload was modified to contain a cleavable valine–citrulline [VC(*S*)] linker, which upon cathepsin B-mediated hydrolysis and subsequent [1,6]-fragmentation of *p*-aminobenzylcarbamate (PAB) releases the free MMAE.^27^ As a control, we used a VC(*R*) linker, which is resistant to cleavage by cathepsin B and other proteases.^28^

Four key considerations were used for the design of the conjugates:

1. The accumulation of albumin in proliferating tumors. For example, 20% of the injected dose per gram of a radio-labeled albumin derivative was shown to selectively accumulate in rats bearing subcutaneous tumors after 24 h.^29^
2. The use of cysteine-selective carbonylacrylic reagents^30,31^ for bioconjugation. Unlike maleimide reagents, the conjugates formed after conjugate addition of carbonylacrylic moieties to cysteine afford conjugates that are stable in plasma and, thus, avoid premature release of the payload.
3. The use of protease-cleavable [VC(*S*)] versus noncleavable [VC(*R*)] linkers^28^ equipped with MMAE provide insights into whether protease-mediated cleavage is required for efficient release of MMAE.
4. The use of an engineered variant of human albumin (Veltis—a technology platform from Albumedix Ltd.) with enhanced binding to the neonatal Fc receptor, which improves FcRn-mediated internalization and recycling^32−34^ and extends serum half-life.^35^

## Results

### Reaction Optimization of Specific Cysteine–Albumin Bioconjugation

We started studying the ability of the carbonylacrylic amide^30,31^**1** to perform bioconjugation reactions with albumin Veltis High Binder II (HBII). The bioconjugation optimization studies with **1** and the protein are described in Table 1. The number of equivalents, temperature, time, ionic strength of the buffer (20 and 50 mM), and pH (7.0 and 8.0) were evaluated. Efficient conversion to the desired product was observed using stoichiometric amounts of **1** at pH 8.0 (NaP_i_, 20 mM), which provided the conjugate in >95% conversion after 30 h at 37 °C (Entry 15, Table 1). Higher amounts of **1** also afforded the conjugate in shorter reactions times (1–4 h) and excellent conversions at 37 °C. For instance, 3 equiv (NaP_i_, pH 8.0, 20 mM, Entry 12, Table 1) and 5 equiv (NaP_i_, pH 8.0, 50 mM, Entry 22, Table 1 and NaP_i_, pH 7.0, 50 mM, Entry 27, Table 1) could also be employed to achieve full conversion (additional details in the Supporting Information).

**Table 1:**
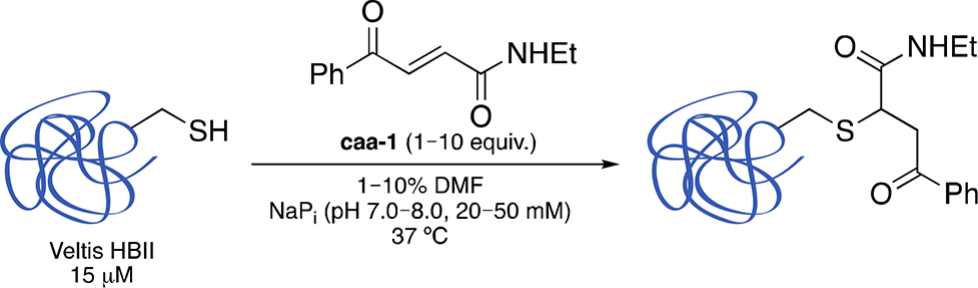
Optimization Studies of the Conjugation Reaction Between Albumin HBII and **1**

entry	equiv of **1**	Veltis HBII (μM)	buffer (mM)	pH	time (h)	conversion (%)^a^	conversion 2nd modification (%)^b^
1	1	15	20	8	1	20	0
2	1	15	20	8	2	30	0
3	1	15	20	8	3	40	0
4	1	15	20	8	4	45	0
5	5	15	20	8	1	70	30
6	5	15	20	8	2	60	40^c^
7	10	15	20	8	1	30	70^c^
8	10	15	20	8	2	20	80^c^
9	2	15	20	8	2	70	0
10	2	15	20	8	3	80	0
11	2	15	20	8	17	80	20
12	3	15	20	8	1	>95	0
13	1	15	20	8	7	55	0
14	1	15	20	8	17	70	0
15	1	15	20	8	30	>95	0
16	1	15	50	8	17	70	0
17	1	15	50	8	36	80	0
18	1	15	20	7	17	40	0
19	1	15	20	7	36	45	0
20	1	15	50	7	17	50	0
21	1	15	50	7	36	70	0
22	5	15	50	8	1	>95	0
23	5	15	20	7	1	70	30
24	5	15	50	7	1	60	0
25	5	15	50	7	2	80	0
26	5	15	50	7	3	>90	0
27	5	15	50	7	4	>95	0

a Conversion toward the single modified Veltis HBII conjugate.

b Conversion toward double addition product.

c Full conversion of starting protein; third modification observed (conversions as judged by HPLC analysis).

### Synthesis of caa-Tagged MMAE and caa-Tagged Fluorophore

**Figure 1:**
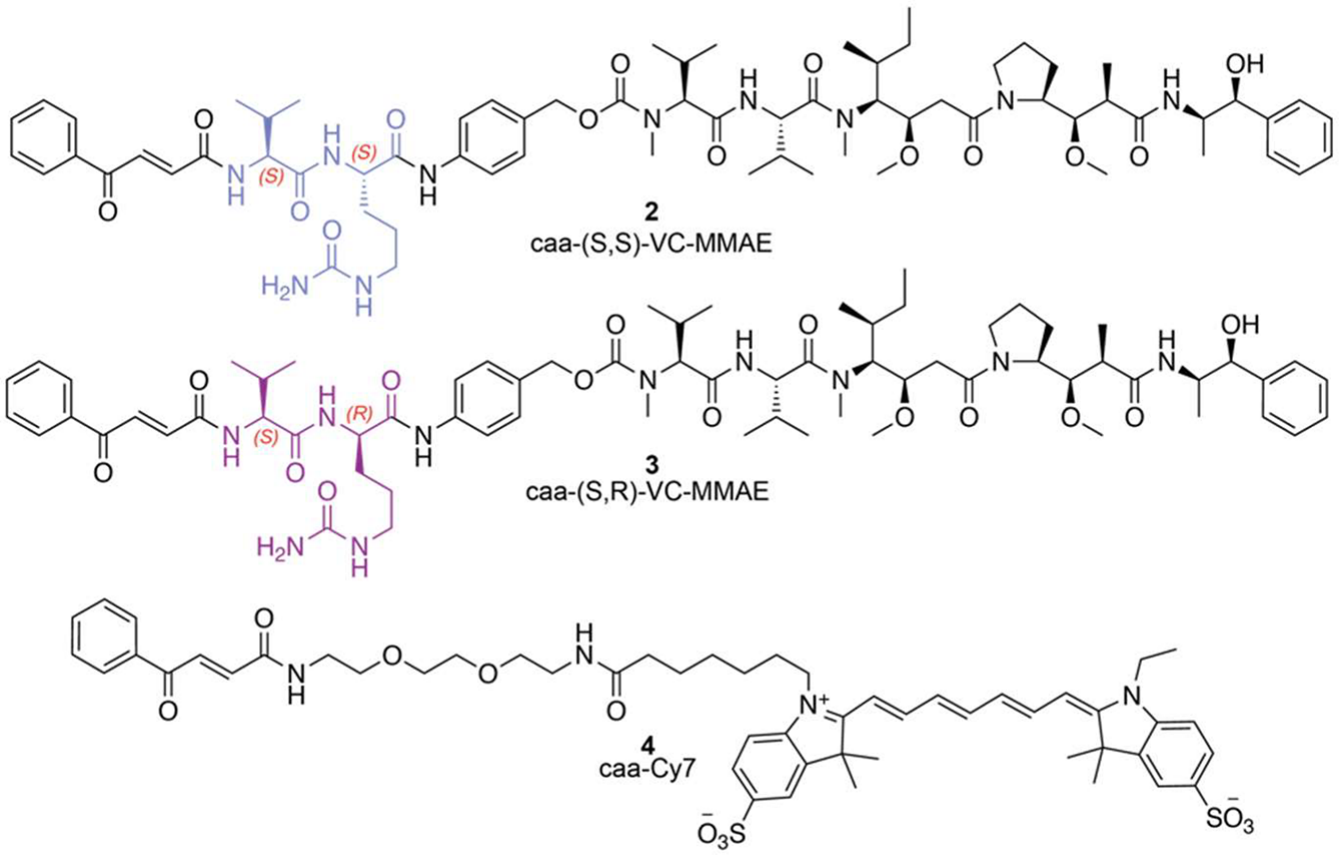
Structures of carbonylacrylic-tagged VC linker + drug used in this study: cleavable caa-(*S*,*S*)-VC-MMAE **2**, noncleavable caa-(*S*,*R*)-VC-MMAE **3**, and caa-Cy7 **4**.

With an optimized method for cysteine-selective bioconjugation, we then started the preparation of two derivatized carbonylacrylic (caa) reagents equipped with a drug payload, followed by chemoselective cysteine modification of albumin HBII (Figure 1). The first example, a caa-tagged MMAE derivative, **2**, was easily synthesized from commercially available MMAE and *trans*-3-benzoylacrylic acid (Figure 1 and Supporting Information for details of the synthesis). A noncleavable linker, caa-VC(*R*)-MMAE, **3**, was also synthesized using the same synthetic strategy (Figure 1 and Supporting Information for details of the synthesis). Incubation of **2** and **3** with cathepsin B at 37 °C resulted in significant enzymatic release of MMAE from **2**, while **3** was stable to enzymatic cleavage (Figures S24 and S25). An example of a carbonylacrylic derivative linked to a cyanine fluorophore, Cy7 **4** (Figure 1), was also prepared for bioimaging studies.

### Construction of Functional Albumin–Drug Conjugates

**Figure 2:**
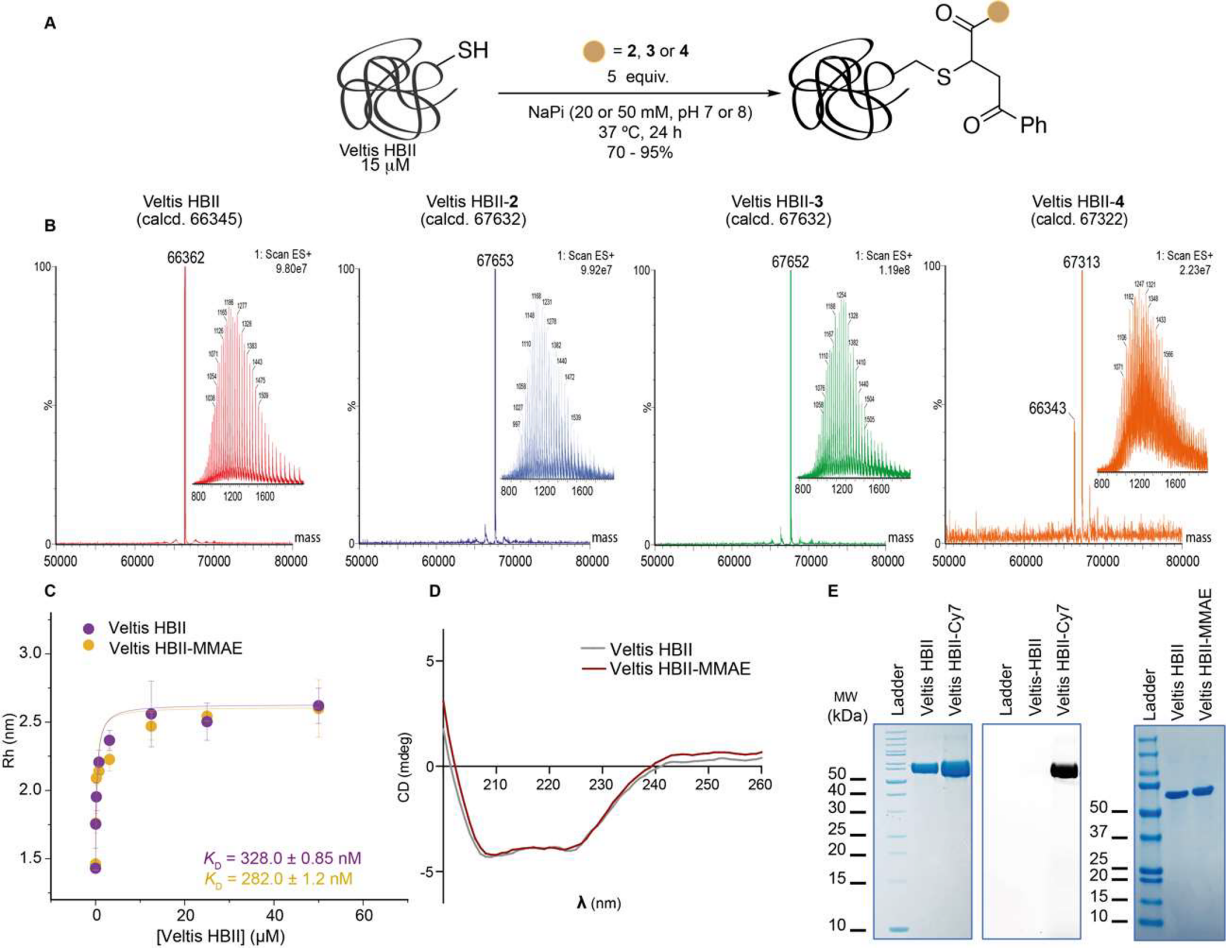
(A) Schematic of the reaction between Veltis HBII with caa-(*S*,*S*)-VC-MMAE, caa-(*S*,*R*)-VC-MMAE, and caa-Cy7. General conditions: 5–10 equiv of the carbonylacrylic reagent in NaP_i_ buffer (pH 7 or 8), 37 °C. (B) Mass spectrometry characterization of cysteine conjugation with **2**, **3**, and **4**. ESI–MS spectra of albumin HBII and the conjugates. (C) Albumin binding to FcRn. Binding curve and resulted dissociation constants (*K*_D_) of FcRn binding with albumin and albumin–MMAE conjugate were measured with microfluidic diffusional sizing. (D) Comparative CD analysis of albumin and albumin–MMAE. (E) SDS-PAGE analysis of albumin–**3** and albumin–**4**. Gels 1 and 3, Coomassie staining. Gel 2, fluorescence.

Having assessed the suitability of **2** and **3** as CatB-cleavable and -noncleavable analogues, respectively, we moved forward toward the synthesis of albumin–drug conjugates. In this direction, we investigated albumin HBII conjugation with the cleavable payload caa-VC(*S*)-MMAE **2** (Figure 2A). Unlike the reaction with the smaller carbonylacrylic amide **1**, we found that the use of one or excess molar equivalents of **2** per cysteine residue did not provide useful conversions to the desired conjugate when the reaction was performed in 50 mM NaP_i_, pH 8.0 after 24 h (Table S1). However, when we treated albumin with 5 equiv of **2**, slightly lowered the pH to 7.0, and changed the ionic strength of the buffer to 20 mM, efficient conversion to the desired product was observed. The best conjugation was obtained using 5 equiv of **2**, NaP_i_ (pH 7.0, 50 mM) for 24 h at 37 °C, and the conversion observed was >95%, as judged by LC-MS (Figure 2B and entry 13, Table S1). The incorporation of the Cy7 fluorophore **4** to albumin HBII was also investigated, and the results are shown in Table S2. In this case, when using 5 equiv of **4** in NaP_i_ buffer (pH 7.0, 20 mM) for 24 h of reaction at 37 °C, only 70% conversion to the fluorophore conjugate was obtained (Figure 2B). The use of higher amounts of **4** led to nonspecific modifications and protein degradation.

Next, we evaluated whether the modified albumin retained its FcRn binding properties. Microfluidic diffusional sizing was used to characterize binding of albumin HBII and conjugate albumin–**2** to the FcRn. The binding was quantified by constructing a binding curve (Figure 2C) by keeping the FcRn concentration constant (42 nM) and exploring different protein concentrations while measuring the hydrodynamic radius of the protein under different conditions. While a small difference in the binding potency was observed, both albumin HBII and its conjugate with **2** showed identical binding properties. This is consistent with data showing that Veltis HBII-caa-VC(S)-MMAE does not present changes in its secondary structural content relative to parent albumin HBII, as determined by circular dichroism (Figure 2D). SDS-PAGE analysis of the native albumin HBII and its conjugates with **4** and **2** is shown in Figure 2E. Treatment of albumin HBII with **4** gave a single new fluorescent band that is consistent with incorporation of the Cy7 fluorophore. This data shows the selectivity of the caa reagents to create homogeneous and functionally active bioconjugates.

### Systemic Treatment of Ovarian Cancer Using Albumin–MMAE Conjugates

**Figure 3:**
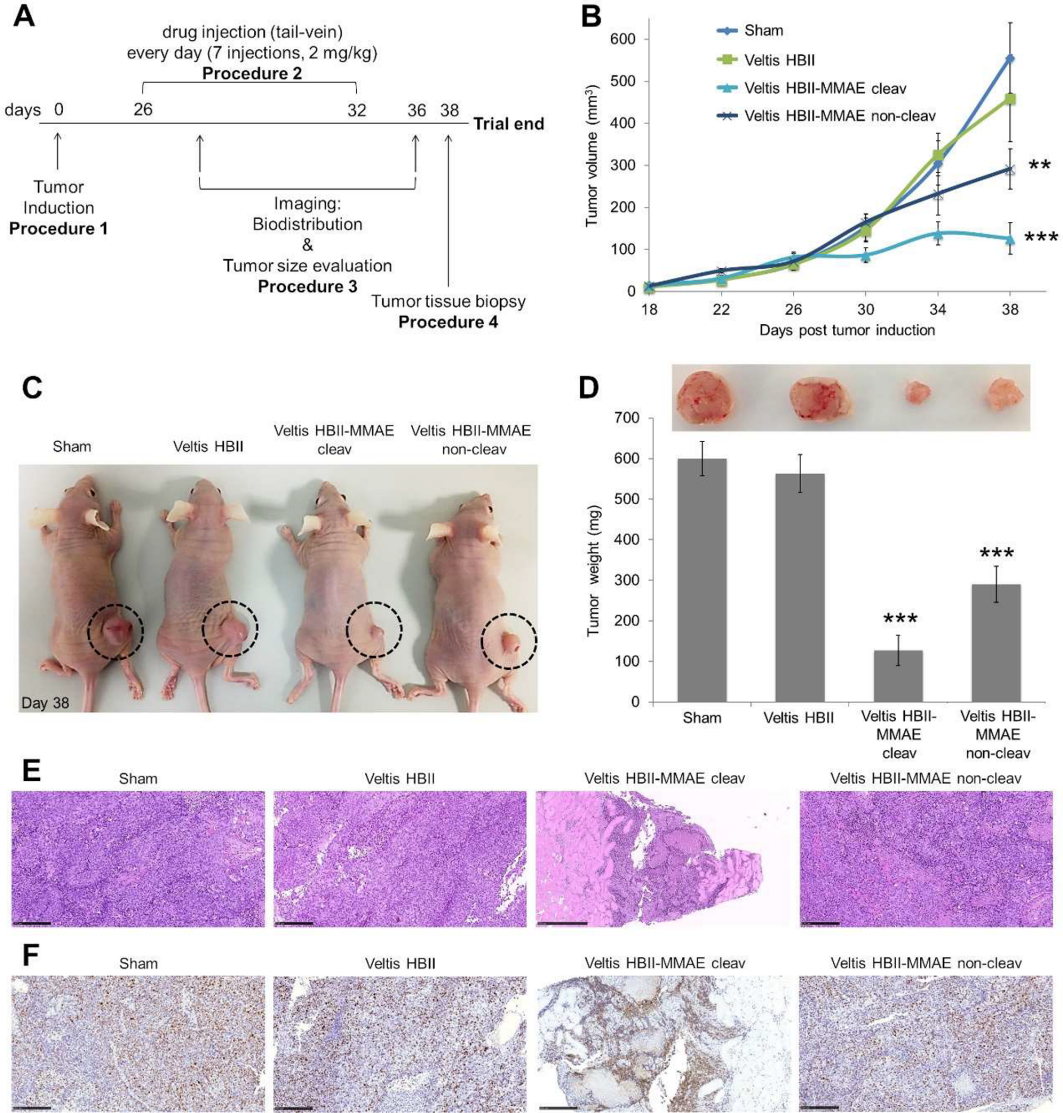
(A) Experiment design for systemic treatment of ovarian cancer using Veltis HBII-caa-VC-MMAE conjugates. (B) Tumor burden in mice treated with Veltis HBII-caa-VC-MMAE conjugates or albumin alone as measured by tumor volume (5 mice per group). For determination of tumor growth, individual tumors were measured (2–3 times per week) using caliper, and tumor volume was calculated by tumor volume (mm^3^) = width × (length^2^)/2 (two-tailed paired Student’s *t* test, **P ≤ 0.001, ***P ≤ 0.0001). (C) Representative images of SK-OV-3 xenograft mice treated with protease-cleavable Veltis HBII-caa-VC(*S*)-MMAE conjugate, noncleavable Veltis HBII-caa-VC(*R*)-MMAE conjugate, albumin alone, or untreated (controls). Data show that the cleavable Veltis HBII-caa-VC(S)-MMAE conjugate treatment successfully reduces tumor volume over a period of 38 days relative to controls in which the tumor grows faster. (D) Mass range of tumors from treated mice when compared with nontreated (sham) (two-tailed paired Student’s *t* test, **P ≤ 0.001, ***P ≤ 0.0001). All values are presented as mean ± SEM. (E) H&E-stained tissue sections and (F) immunohistochemical evaluation of Ki67 for tumors treated with Veltis HBII-caa-VC(*S*)-MMAE, Veltis HBII-caa-VC(*R*)-MMAE, or albumin alone.

The capability of the synthesized albumin conjugates **2** and **3** to shrink tumors was evaluated in an ovarian cancer mouse model following systemic administration by intravenous injection of the conjugates (Figure 3A). Briefly, subcutaneous tumors were induced in female athymic nude mice by injection of a high-grade serous ovarian adenocarcinoma cell line (SK-OV-3 cells) with epithelial-like morphology, which recapitulates a human ovarian adenocarcinoma. When these mice were treated with albumin HBII conjugated to MMAE via the protease-cleavable dipeptide [VC(*S*)], a highly significant tumor size reduction was achieved (Figure 3B,C). Strikingly, the administration of 2 mg/kg of the conjugate resulted in tumor abrogation (approximately 86%, ***P ≤ 0.0001), as well as tumor mass reduction (Figure 3D, approximately 80%, ***P ≤ 0.0001).

Concerning histopathology analysis, the tumors in the sham, albumin, and the noncleavable Veltis HBII-caa-VC(*R*)-MMAE-treated groups show high cellular density composed of a poorly differentiated population of neoplastic cells arranged in cords and nests separated by fusiform cells/fibroblasts. The tumor cells show a high mitotic index (3 to 5 mitotic figures per high-power fields). There are also multifocal to coalescing areas of necrosis. In contrast, the tumors from the cleavable Veltis HBII-caa-VC(S)-MMAE treatment group show low infiltration by neoplastic cells into muscle tissue. No mitotic figures were seen in these tumors. There are some multifocal areas with clusters of necrotic myocytes (Figure 3E). Moreover, immunohistochemical analysis showed that Ki67 (a cellular marker exclusively linked with cell proliferation) expression was reduced following treatment with cleavable albumin–MMAE (Figure 3F). This data clearly shows that tumor progression was extensively impaired in tumor-bearing mice treated with the protease-cleavable conjugate Veltis HBII-caa-VC(*S*)-MMAE but not with noncleavable conjugate Veltis HBII-caa-VC(R)-MMAE.

To validate safety, 38 days post-tumor induction and 12 days after conjugates administration, major organs were harvested from mice and H&E-stained for routine pathological analysis (Figure S2). H&E staining showed that *in vivo* administration of all the tested compounds did not cause any damage in several organs (i.e., lung, liver, kidney, spleen, heart, and intestines) when compared with the control group (sham, i.e., saline solution injection).

The safety of all drugs was also confirmed by monitoring body weight as a proxy for tolerability. No *in vivo* toxicity or other physiological complications were observed in all of the animal groups for 12 days postdrug exposure, as indicated by the maintenance of stable body weight (Figure S3), thereby suggesting that none of the drug treatments were toxic.

**Figure 4:**
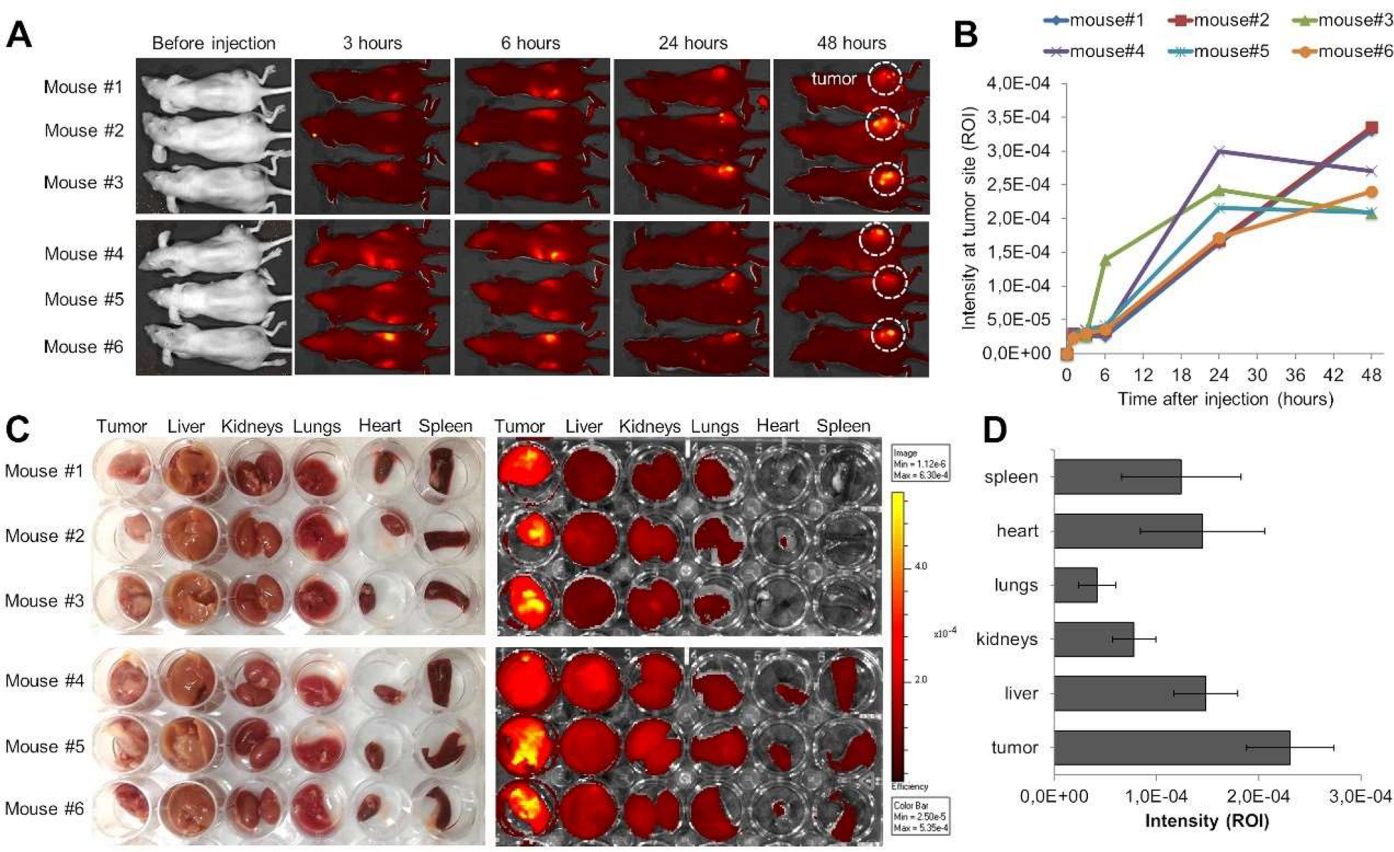
(A) Live imaging of athymic nude mice with ovarian tumor xenografts implanted in the right-side flank (6 mice per group). (B) Fluorescence intensity signal at tumor site for 1.5, 3, 6, 24, and 48 h postalbumin conjugate injection. (C) *Ex vivo* images of tumors and whole body organs (liver, kidneys, spleen, heart, and lungs) are also presented. (D) Fluorescence intensity signal for tumors and whole body organs. All values are presented as mean ± SEM.

Biodistribution of albumin conjugates in primary tumors and major organs were tracked fluorescently via a live imaging system up to 48 h after IV injection of the albumin conjugates Cy7. Live imaging of mice (Figure 4A,B) and *ex vivo* fluorescent images of ovarian tumors and major organs (liver, kidneys, lungs, heart, and spleen) revealed that Cy7-labeled albumin was able to extensively accumulate in tumors 24 h postadministration (Figure 4C,D). This confirms the capacity of these conjugates to leak through the tumor vasculature and penetrate and accumulate in the tumor tissue efficiently. No signs of inflammation, nor changes in body weight, were observed before or after tumor induction or conjugate administration (tail vein injection), which suggests that albumin conjugates are biocompatible with no associated toxicity or side effects.

## Conclusions

In conclusion, the use of an engineered version of albumin with high affinity to both human and mouse FcRn^35^ combined with cathepsin B cleavable (*S,S*-valine–citrulline) and noncleavable (*S,R*-valine–citrulline) linkers equipped with MMAE suggest that cathepsin B is required to achieve efficient antitumor activity. In our study, the *S,R*-valine–citrulline noncleavable linker only exhibited moderate activity *in vivo* (half potency relative to the cleavable *S,S*-valine–citrulline linker), which likely resulted from a toxic MMAE–catabolite generated during lysosomal activity. We also demonstrated the preferential tumor biodistribution of our homogeneous albumin conjugates, as well as their biocompatibility, with no associated toxicity or side effects. These results suggest that the combination of enhanced Fc binding provided by the engineered albumin used in this study with the use of a protease cleavable linker is an efficient targeted drug delivery strategy to treat solid tumors.

## Supporting Information Available

Supporting figures, detailed methods, and additional data (PDF)
