# Supplementary material for: Cathepsin B Processing Is Required for the In Vivo Efficacy of Albumin–Drug Conjugates
Source: Bioconjug Chem. 2024 Feb 12;35(2):132–9. doi: 10.1021/acs.bioconjchem.3c00478 (PMC10885003; doi:10.1021/acs.bioconjchem.3c00478)
Supplement: Supplementary file 1 — bc3c00478_si_001.pdf [file bc3c00478_si_001.pdf]

SUPPORTING INFORMATION

Cathepsin B Processing is Required for the *in vivo* Efficacy of Albumin-Drug Conjugates

Barbara Bernardim[1#], Joao Conde[2#], Tuuli Hakala[1], Julie Becher[1], Mary Canzano[1], Aldrin V. Vasco[1], Tuomas P. J. Knowles[1], Jason Cameron[3], and Gonçalo J. L. Bernardes[1,2*]

[1] Yusuf Hamied Department of Chemistry, University of Cambridge, Lensfield Road, CB2 1EW, Cambridge, UK

[2] Instituto de Medicina Molecular João Lobo Antunes, Faculdade de Medicina, Universidade de Lisboa Avenida Professor Egas Moniz,1649-028, Lisboa, Portugal

[3] Albumedix Ltd, Mabel Street, Nottingham, NG2 3ED, UK

[#]These authors contributed equally to this work.

Correspondence should be addressed to G.J.L.B.: E-mail: gb453@cam.ac.uk

TABLE OF CONTENTS

1. Supporting figures S3
2. Procedure for the preparation of caa-(S,S)-VC-PABA-MMAE (2) S5
3. Procedure for the preparation of caa-(S,R)-VC-MMAE (3) S9
4. Procedure for the preparation of caa-Cy7 (4) S12
5. General procedure for bioconjugation of proteins S15
6. Modification of Veltis S17
7. Scale-up experiment with caa-(S,S)-VC-MMAE S22
8. Scale-up experiment with caa-(S,R)-VC-MMAE S26
9. Optimisation of the reaction with caa-Cy7 S28
10. Modification of Veltis with Cy7 fluorophore (small scale) S29
11. Stability in plasma S30
12. Circular dichroism analysis of Veltis S31
13. SDS-PAGE analysis S31
14. Measuring hydrodynamic radiuses and binding curves S32
15. Development of ovarian cancer mice model and albumin-conjugates administration S33
16. Biodistribution analysis of albumin-conjugates S33
17. Statistics S33
18. Enzymatic cleavage assay for caa-(S,S)-VC-MMAE and caa-(S,R)-VC-MMAE in the presence of Cathepsin B S34
19. General LCMS Conditions S38
20. References S38

1. Supporting figures

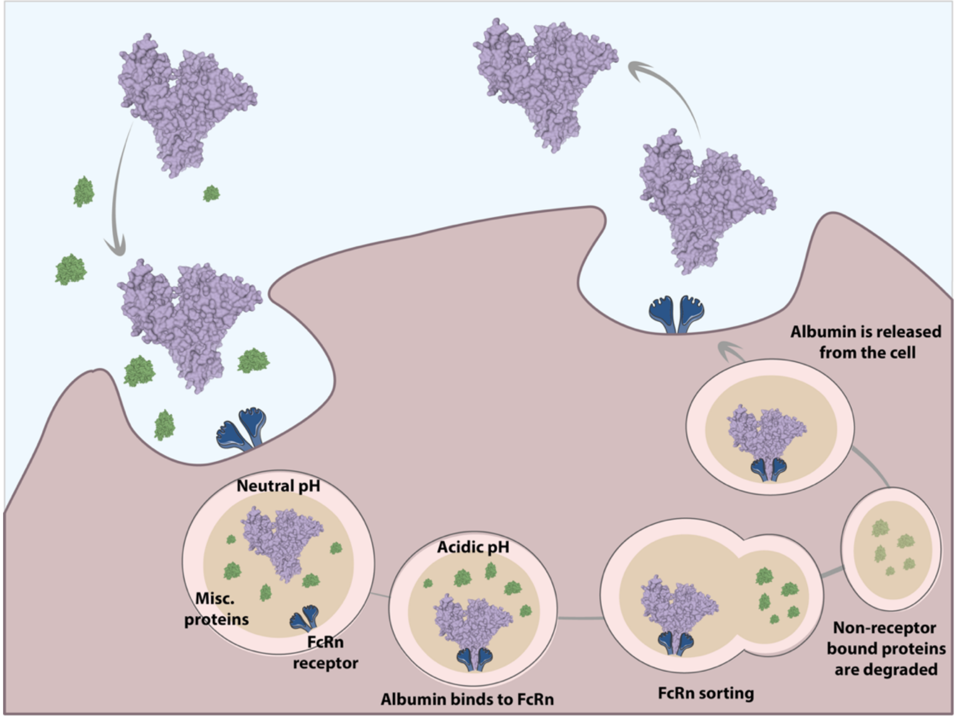

Figure S1. Mechanism of albumin FcRn-mediated recycling.

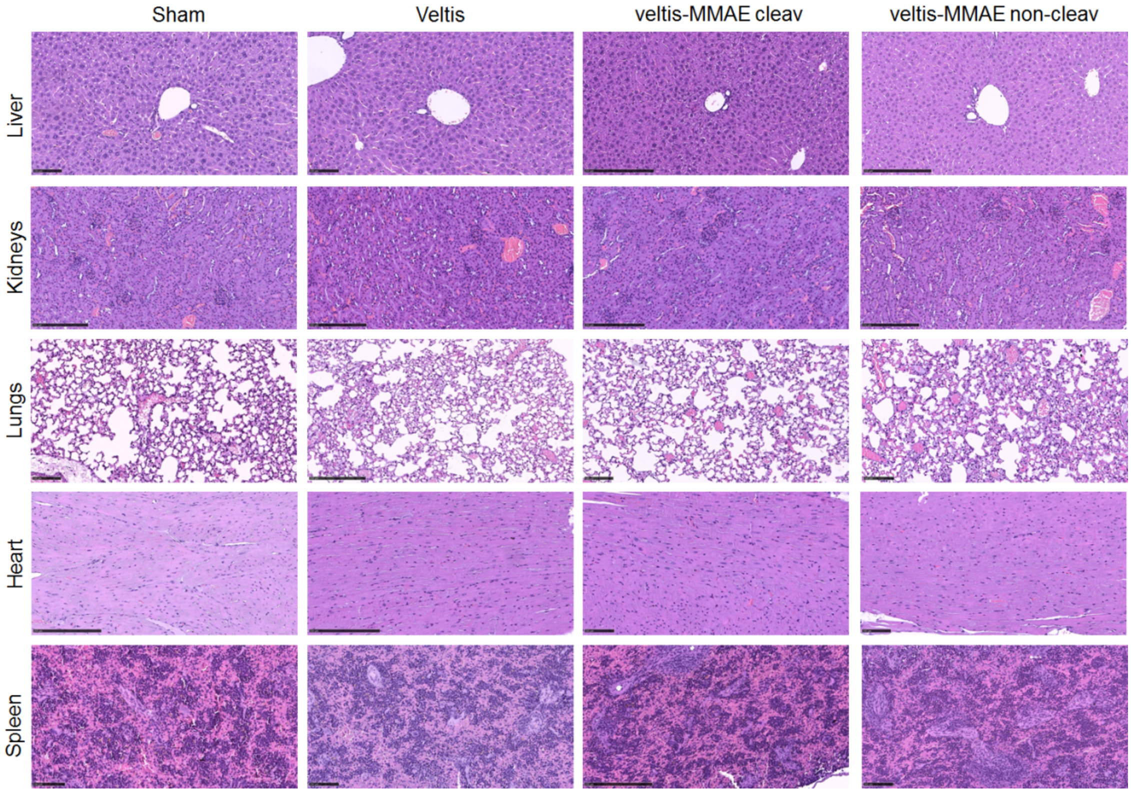

Figure S2. Safety assessment. H&E stained tissue sections of the organs (i.e. lung, liver, kidney, spleen, heart) collected from mice 38 days post tumour induction and 12 days after conjugates administration.

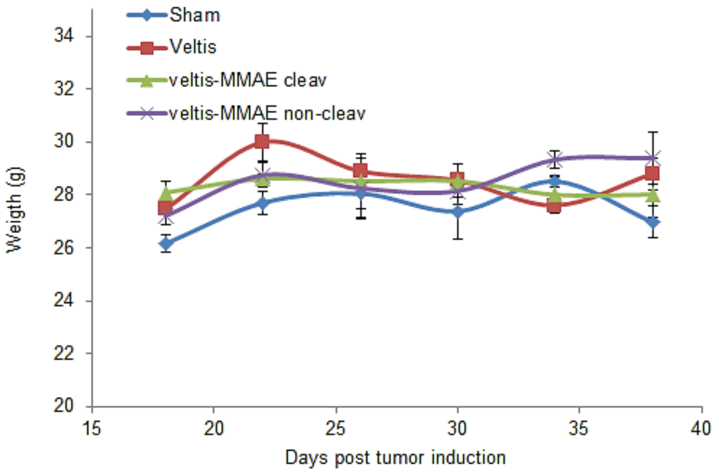

Figure S3. The safety of the Veltis conjugates was confirmed by monitoring body weight as a proxy for tolerability. Body weight assessment was performed on all the animal groups during 38 days after ovarian tumours induction and 20 days after conjugates injection. Body weight depicted as the mean of each treatment group. No decrease or changes in body weight were found for all treatment mice groups (n= 5).

2. Procedure for the preparation of caa-(S,S)-VC-PABA-MMAE (2)

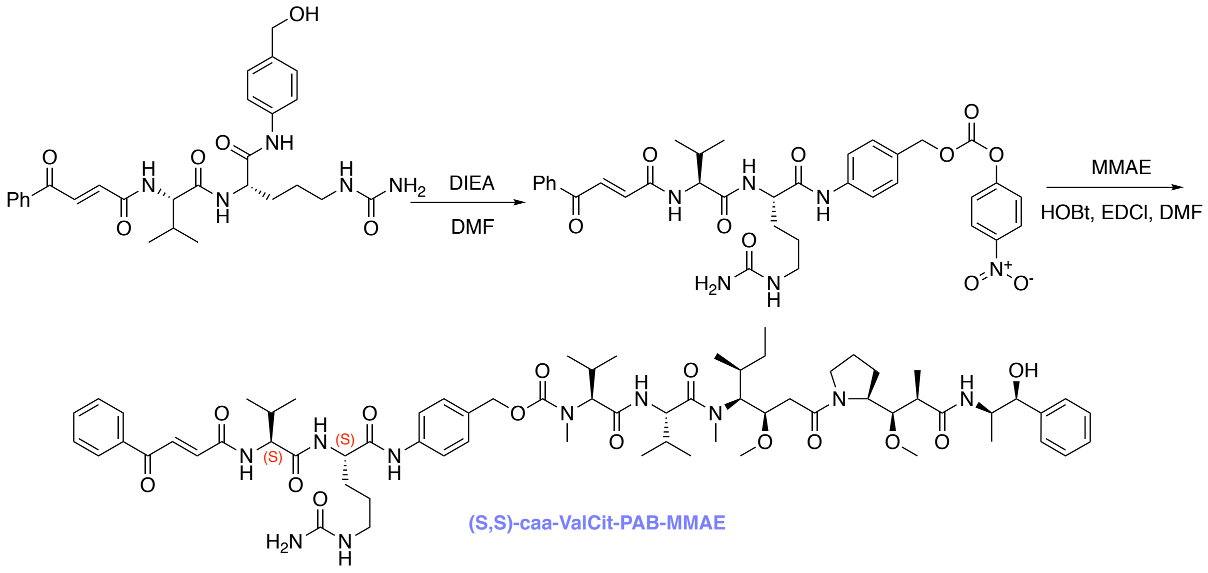

SAFETY NOTE: *Auristatins are potent cytotoxic compounds and must be handled with extreme care and following strict safety measurements. For a guideline consult "Safe handling of cytotoxics: guideline recommendations". Curr Oncol. 2015 Feb;22(1): e27-37. doi: 10.3747/co.21.2151. PMID: 25684994; PMCID: PMC4324350.*

Synthesis of caa-(S,S)-VC-PABA-PNP

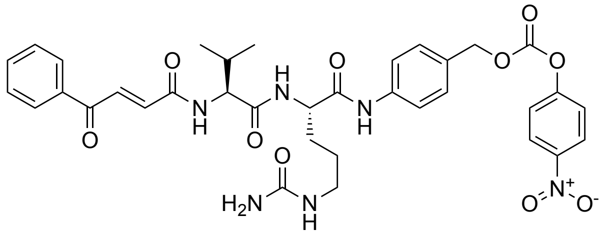

To a solution of caa-(S,S)-VC-PABA-OH (1.5 g, 2.79 mmol, 1.0 equiv.) and bis(4-nitrophenyl) carbonate (3.40 g, 11.16 mmol, 4.0 equiv.) in DMF (10 mL) was added DIEA (2.16 g, 16.74 mmol, 2.92 mL, 6.0 equiv.). The mixture was stirred at 20 °C for 1 h. LC-MS showed starting material was consumed completely and one main peak with desired m/z. The reaction mixture was concentrated under reduced pressure to remove solvent to give a residue. The residue was purified by prep-HPLC (neutral condition). caa-(S,S)-VC-PABA-PNP (270 mg, 384.23 umol, 14% yield) was obtained as a white solid.

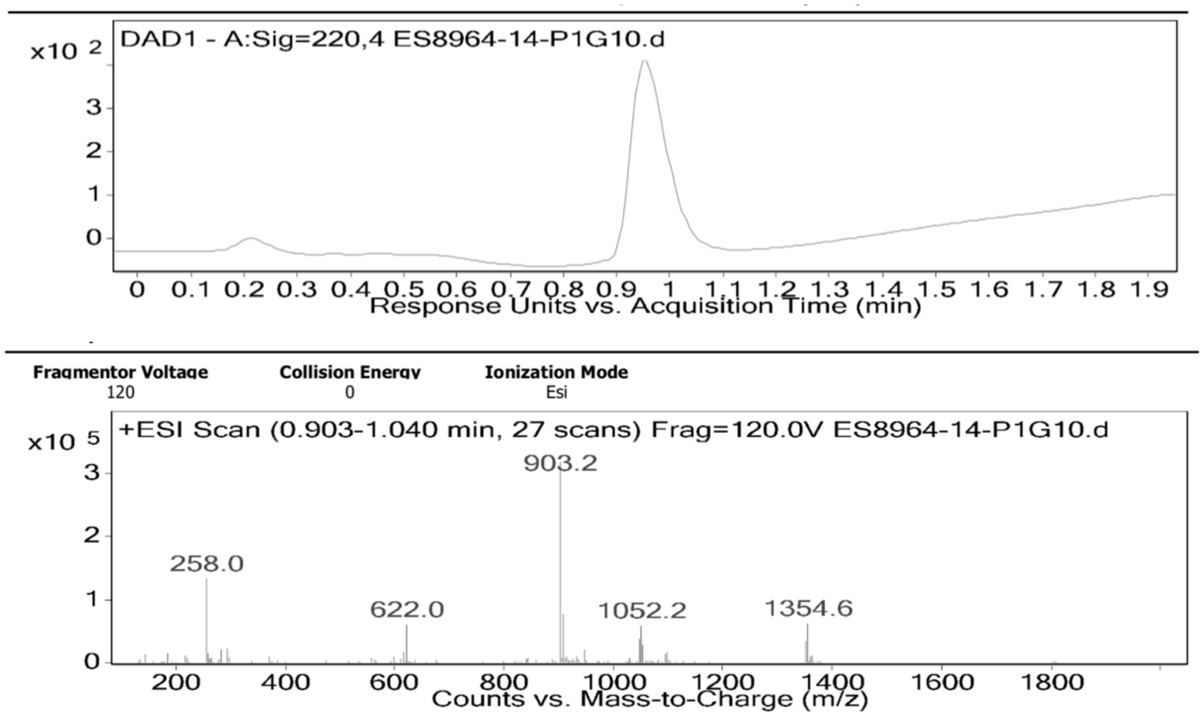

Figure S4. LC-MS spectra of compound caa-(S,S)-VC-PABA-PNP.

Synthesis of caa-(S,S)-VC-MMAE

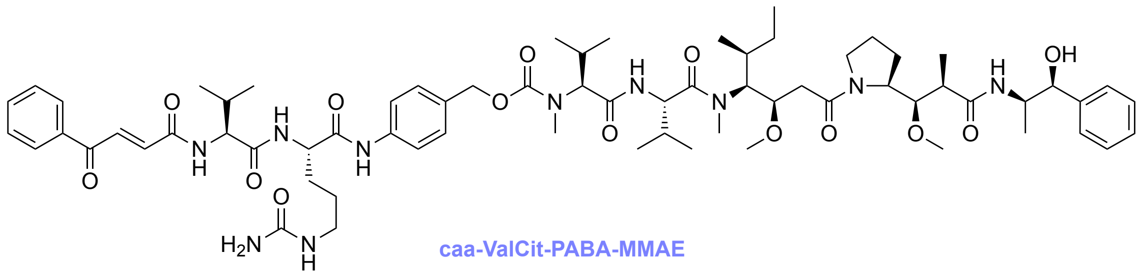

To a solution of caa-(S,S)-VC-PABA-PNP (0.260 g, 370.00 umol, 1.0 equiv.) in DMF (1 mL) was added HOBt (50.00 mg, 370.00 umol, 1.0 equiv.), DIEA (143.46 mg, 1.11 mmol, 193.34 uL, 3.0 equiv.) and MMAE (265.65 mg, 370.00 umol, 1.0 equiv.). The mixture was stirred at 20 °C for 16 h. LC-MS showed caa-(S,S)-VC-PABA-PNP was consumed completely and one main peak with desired m/z. The reaction mixture was concentrated under reduced pressure to remove solvent to give a residue. The residue was purified by prep-HPLC (neutral condition). caa-(S,S)-VC-MMAE (0.186 g, 145.13 umol, 39 % yield) was obtained as a white solid. HRMS: (m/z) $[M + Na]^+$ calcd. for $C_{68}H_{100}N_{10}NaO_{14}$, 1303.7313; found 1303.7317. ^{1}H-NMR (400 MHz, DMSO-d_6) δ ppm 9.99 (brs, 1H amide), 8.66 (d, J= 8.6 Hz, 1H, amide), 8.31 (d, J= 7.5 Hz, 1H, amide), 8.05 – 7.97 (m, 2H, amide,

Ar), 7.89 (d, J=7.9 Hz, 0.5H), 7.76 (d, J=15.3, 1H, HC=CH), 7.74 – 7.69 (m, 1H, Ar), 7.64 – 7.56 (m, 5H, Ar), 7.32-7.22 (m, 7H, Ar, HC=CH), 7.20 – 7.15 (m, 1H, Ar), 5.99 (brs, 1H), 5.39 (brs, 2H), 5.12-4.96 (m, 2H), 4.74-4.63 (m, 1H), 4.49-4.48 (m, 1H), 4.44 – 4.37 (m, 2H), 4.26 (pt, J = 11.4 Hz, 1H), 4.04 – 3.92 (m, 2H), 3.78 (dd, J=9.4, 2.3 Hz, 0.5H), 3.59 – 3.53 (m, 2H), 3.24-3.23 (m, 4H), 3.20 (s, 2H), 3.18 (s, 1H), 3.12 (s, 2H), 3.06 – 3.02 (m, 2H), 2.97 (s, 2H), 2.88-2.78 (m, 3H), 2.41 (m, 1H), 2.27 (m, 1H), 2.18 – 1.88 (m, 4H), 1.89 – 1.67 (m, 4H), 1.48 (m, 6H), 1.0-0.95 (m, 7H), 0.92 – 0.71 (m, 26H); ^{13}C-NMR (75 MHz, DMSO d-6) δ ppm of characteristic carbon signal: 190.5 (PhC=O), 164.0 (CH=CHCONH$_2$), 159.3 (C=O carbamate).

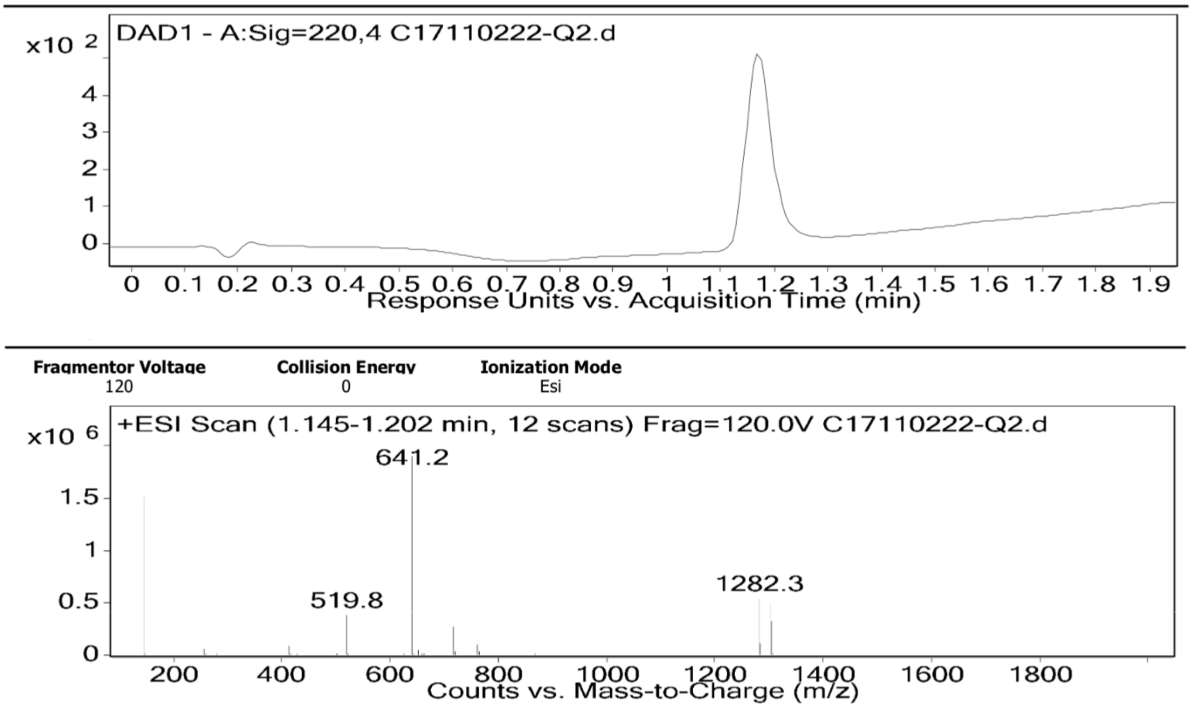

Figure S5. LC-MS spectra of caa-(S,S)-VC-MMAE.

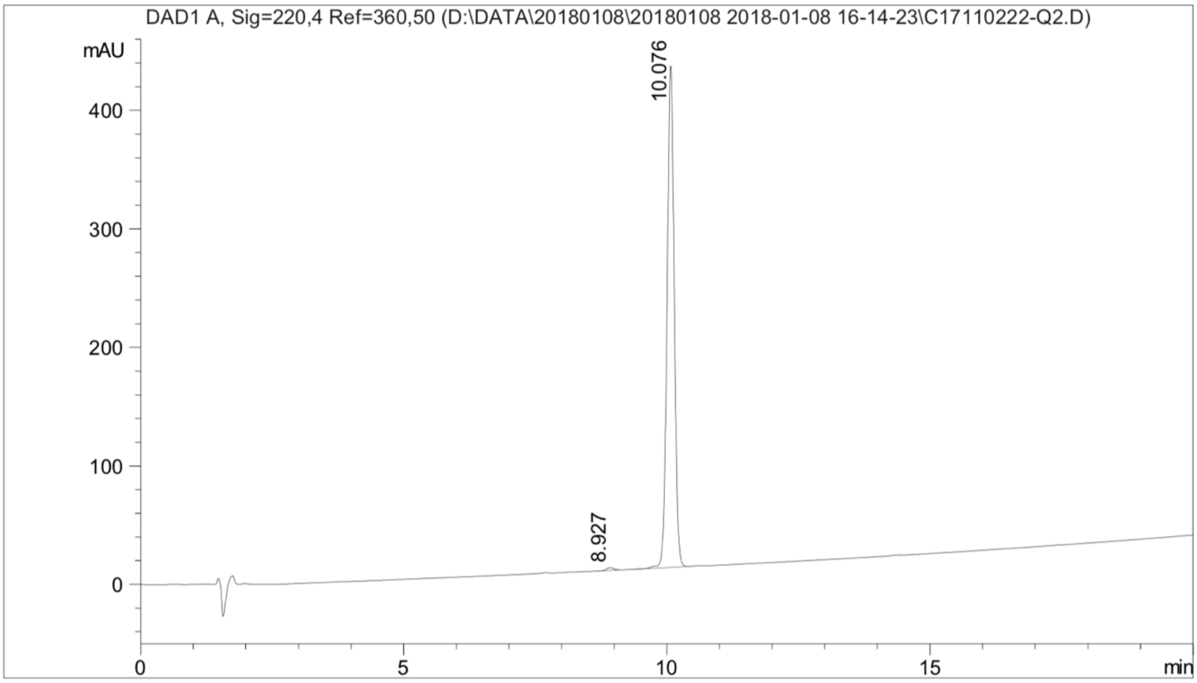

Figure S6. HPLC spectra of caa-(S,S)-VC-MMAE.

3. Procedure for the preparation of caa-(S,R)-VC-MMAE (3)

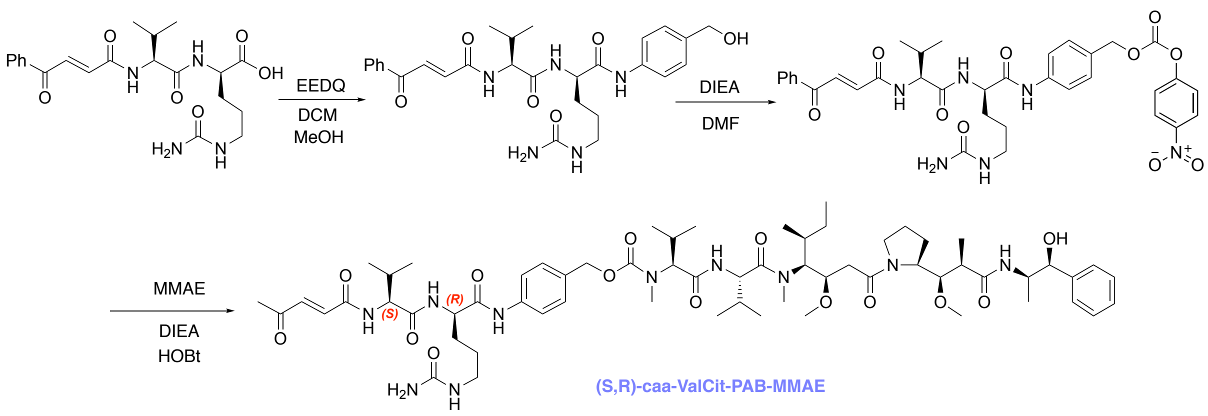

Synthesis of caa-(S,R)-VC-PABA-OH

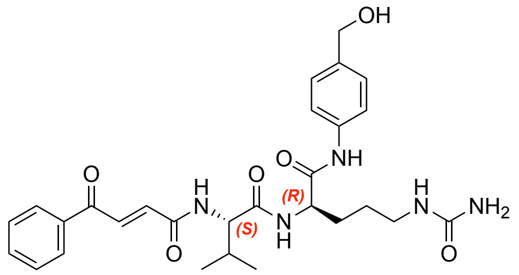

To a solution of caa-(S,R)-VC-OH (4 g, 9.25 mmol, 1.0 equiv.) and (4-aminophenyl)methanol (1.37 g, 11.10 mmol, 1.2 equiv.) in DCM (12 mL), MeOH (6 mL) and DMF (3 mL) was added EEDQ (4.57 g, 18.50 mmol, 2.0 equiv.) The mixture was stirred at 40 °C for 20 h. LC-MS showed starting material was consumed completely and one main peak with desired m/z. The MeOH-mixture was concentrated under reduced pressure to remove solvent. The residue was purified by prep-HPLC (neutral condition). caa-(S,R)-VC-PABA-OH (1.5 g, 2.79 mmol, 30% yield) was obtained as a yellow oil.

Synthesis of caa-(S,R)-VC-PABA-PNP

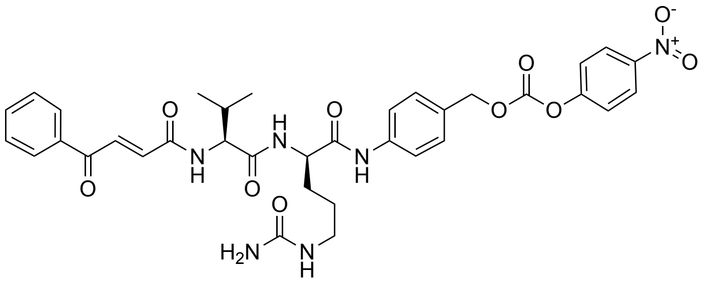

To a solution of caa-(S,R)-VC-PABA-OH (1.5 g, 2.79 mmol, 1.0 equiv.) and bis(4-nitrophenyl) carbonate (3.40 g, 11.16 mmol, 4.0 equiv.) in DMF (10 mL) was added DIEA (2.16 g, 16.74 mmol, 2.92 mL, 6.0 equiv.). The mixture was stirred at 20 °C for 1 h. LC-MS showed starting material was consumed completely and one main peak with desired m/z. The reaction mixture was concentrated under reduced pressure to remove solvent to give a residue. The residue was purified by prep-HPLC (neutral condition). caa-(S,R)-VC-PABA-PNP (270 mg, 384.23 umol, 14% yield) was obtained as a white solid.

Synthesis of caa-(S,R)-VC-MMAE

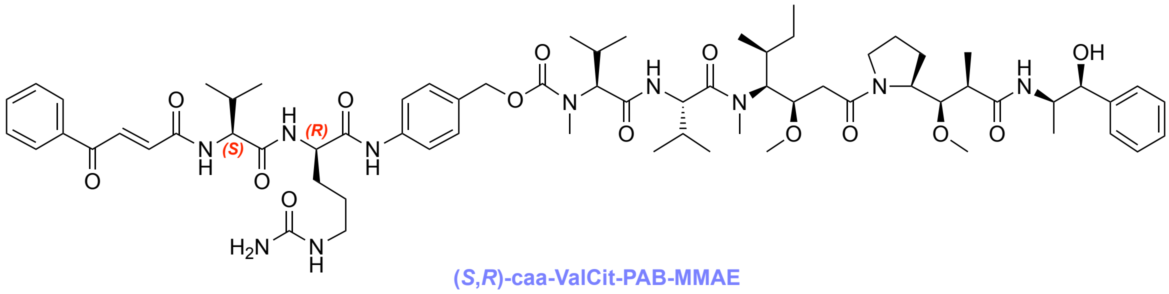

To a solution of caa-(S,R)-VC-PABA-PNP (0.360 g, 409.84 umol, 1.0 equiv.) in DMF (5 mL) was added HOBt (71.99 mg, 532.80 umol, 1.3 equiv.) and DIEA (158.91 mg, 1.23 mmol, 214.16 uL, 3.0 equiv.) was added MMAE (294.26 mg, 409.84 umol, 1.0 equiv.). The mixture was stirred at 20 °C for 16 h. LC-MS showed Compound 6 was consumed completely and one main peak with desired m/z. The reaction mixture was concentrated under reduced pressure to remove solvent to give a residue. The residue was purified by prep-HPLC (neutral condition). caa-(S,R)-VC-MMAE (128.9 mg, 100.58 umol, 24% yield) was obtained as a white solid.

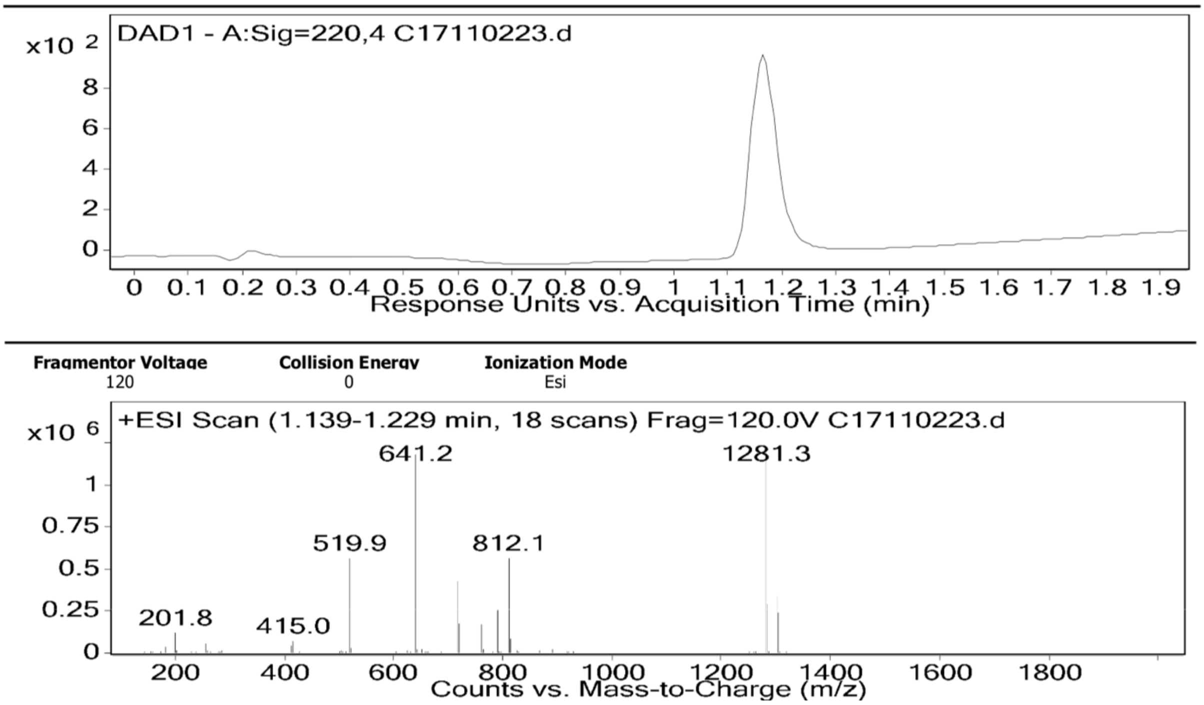

Figure S7. LC-MS spectra of caa-(S,R)-VC-MMAE.

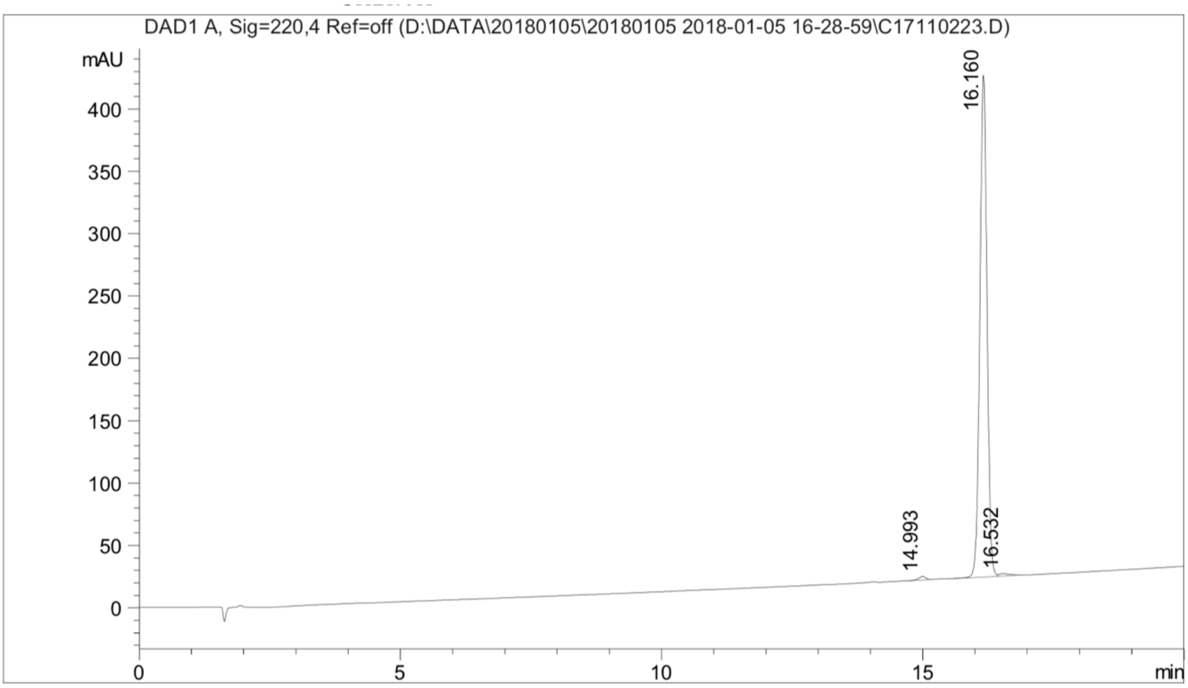

Figure S8. HPLC spectra of caa-(S,R)-VC-MMAE.

4. Procedure for preparation of caa-Cy7 (4)

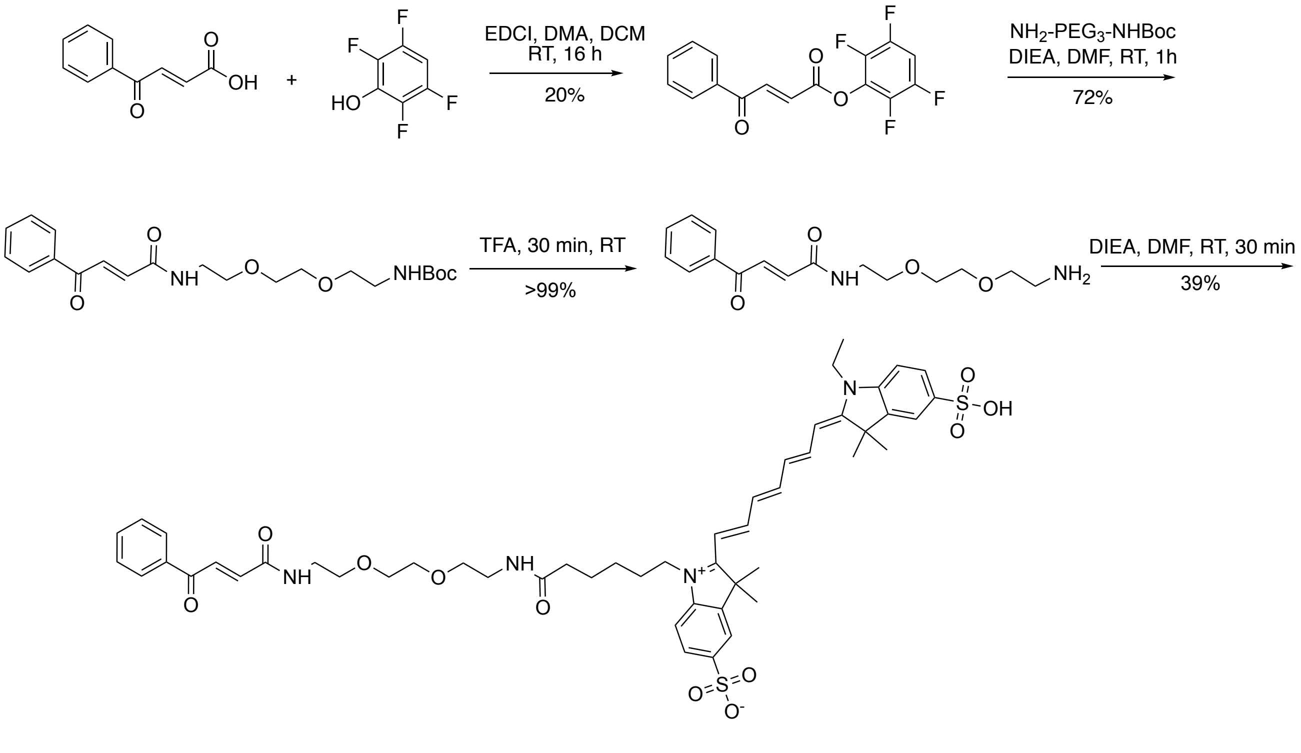

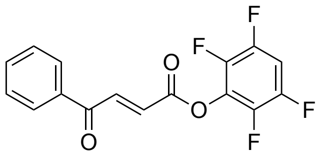

To a solution of the commercially available (E)-benzoylacrylic acid (565.61 mg, 3.41 mmol, 2.0 equiv.) in DMA (6 mL) and DCM (3 mL) was added EDCI (652.90 mg, 3.41 mmol, 2.0 equiv.) followed by 2,3,5,6-tetrafluorophenol. The mixture was stirred at 20 °C for 16 h. TLC showed the acid completely consumed. The reaction mixture was concentrated under reduced pressure to remove solvent to give a residue that was purified by flash chromatography. The activated carbonylacrylic-TFP (0.110 g, 339.27 umol, 20% yield) was obtained as a yellow solid.

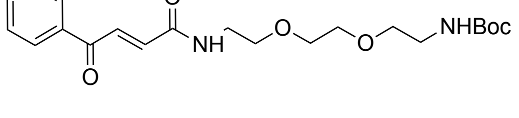

To a solution of caa-TFP (0.110 g, 339.27 umol, 1.0 equiv.) in DMF (2 mL) was added DIEA (87.70 mg, 678.54 umol, 118.19 uL, 2.0 equiv.) and tert-butyl N-[2-[2-(2-aminoethoxy)ethoxy]ethyl]carbamate (75.82 mg, 305.34 umol, 0.9 equiv.) slowly. The mixture was stirred at 20 °C for 1 h. LC-MS showed starting material was consumed completely and one main peak with desired m/z. The reaction was directly purified by prep-HPLC (neutral condition). caa-PEG-Boc (0.100 g, 246.02 umol, 73% yield) was obtained as yellow oil.

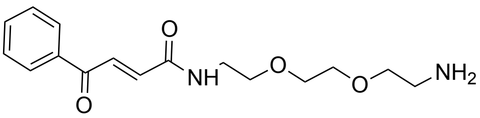

To a solution of caa-PEG-Boc (0.100 g, 246.02 umol, 1.0 equiv.) in DCM (0.9 mL) was added TFA (1.54 g, 13.51 mmol, 1000.00 uL, 54.90 equiv.). The mixture was stirred at 20 °C for 30 min. LC-MS showed caa-PEG-Boc was consumed completely and one main peak with desired m/z. The reaction mixture was concentrated under reduced pressure to remove solvent to give a residue. The purple oil of the crude product (0.100 g, crude, TFA) was used into the next step without further purification.

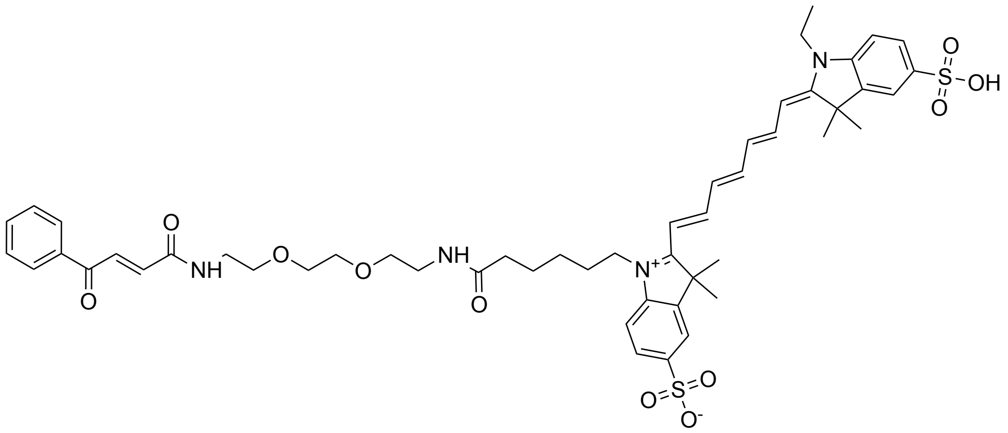

To a solution of deprotected amine (0.005 g, 6.41 umol, 1.0 equiv.) in DMF (0.5 mL) was added DIEA (6.63 mg, 51.29 umol, 8.93 uL, 8.0 equiv.) and reactant NHS ester (9.82 mg, 32.05 umol, 5 equiv.). The mixture was stirred at 20 °C for 2 h. LC-MS showed reactant NHS ester was consumed completely and one main peak with desired m/z was detected. The reaction was directly purified by prep-HPLC (AcOH condition) to give (2.6 mg, 2.50 umol, 39% yield, 93.3% purity) as a blue solid.

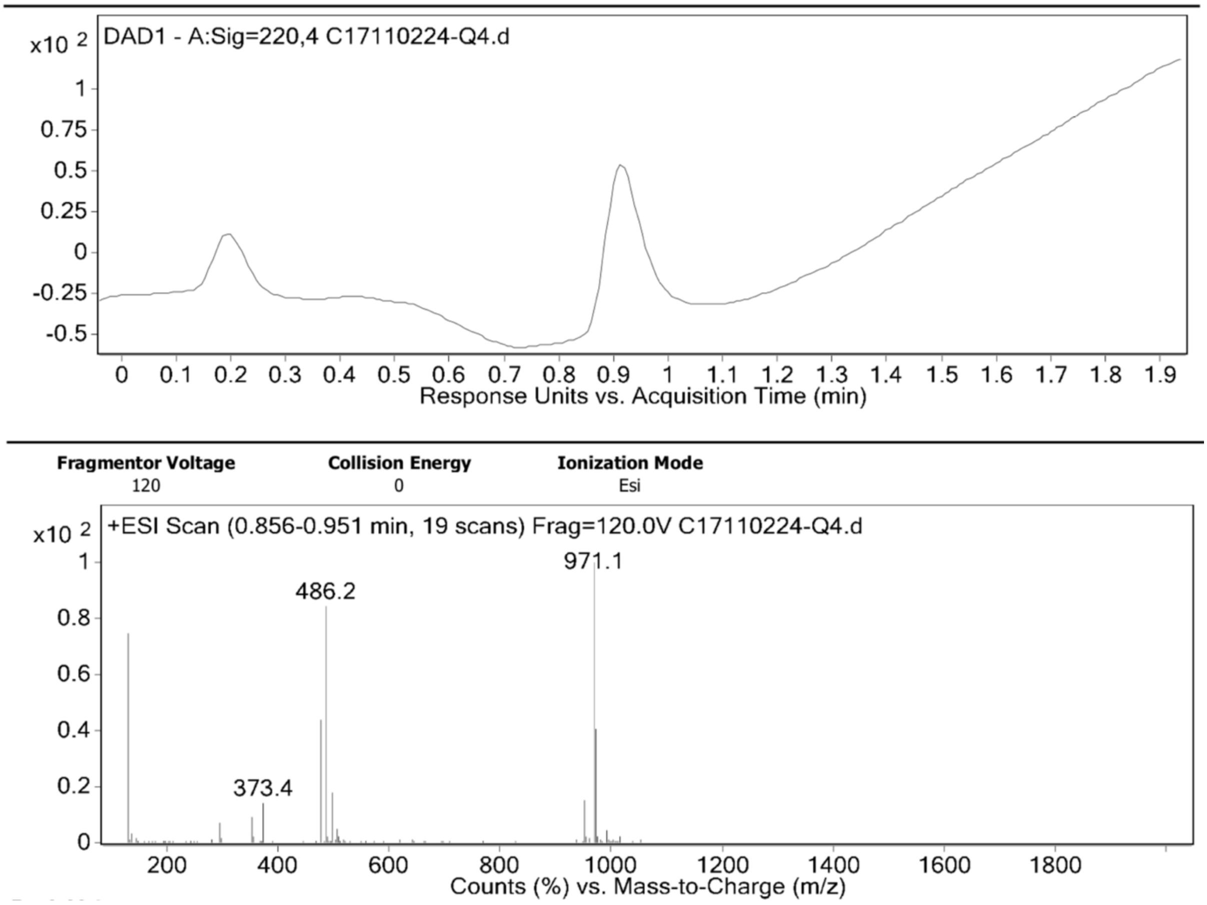

Figure S9. LC-MS spectra of caa-Cy7.

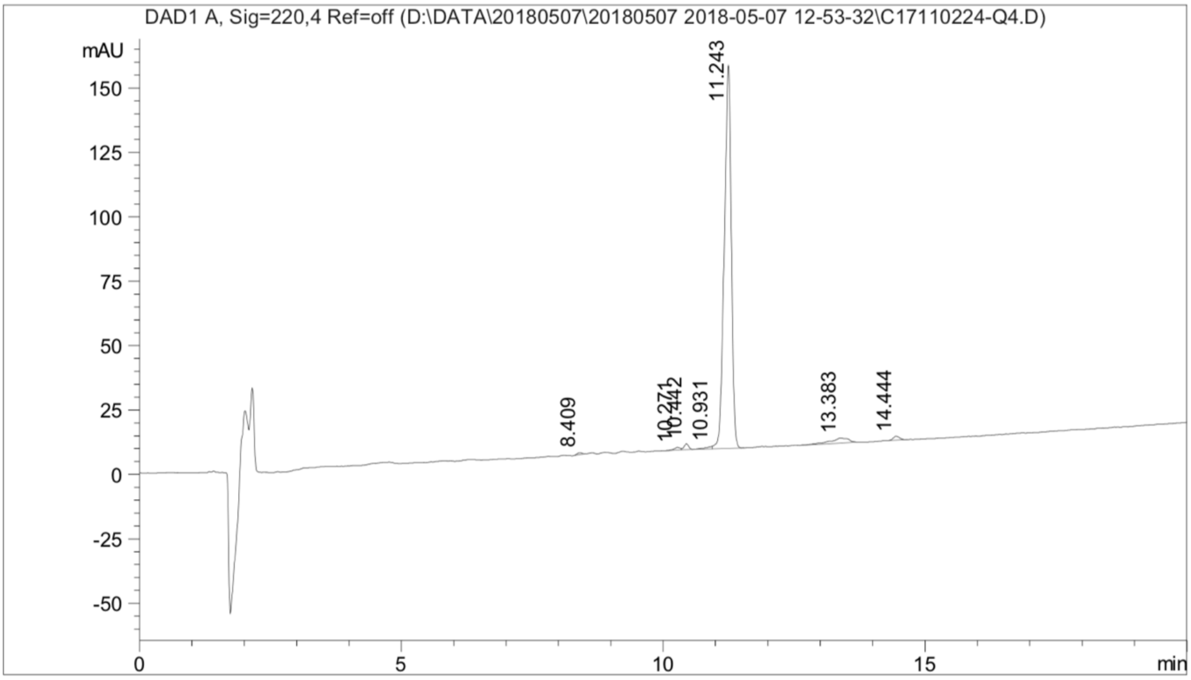

Figure S10. HPLC spectra of caa-Cy7.

5. General procedure for bioconjugation of proteins

A typical analysis of a conjugation reaction by LC–MS is described. The total ion chromatogram, combined ion series and deconvoluted spectra are shown for the starting material and the product of the reaction. Identical analyses were carried out for all the conjugation reactions performed in this work.

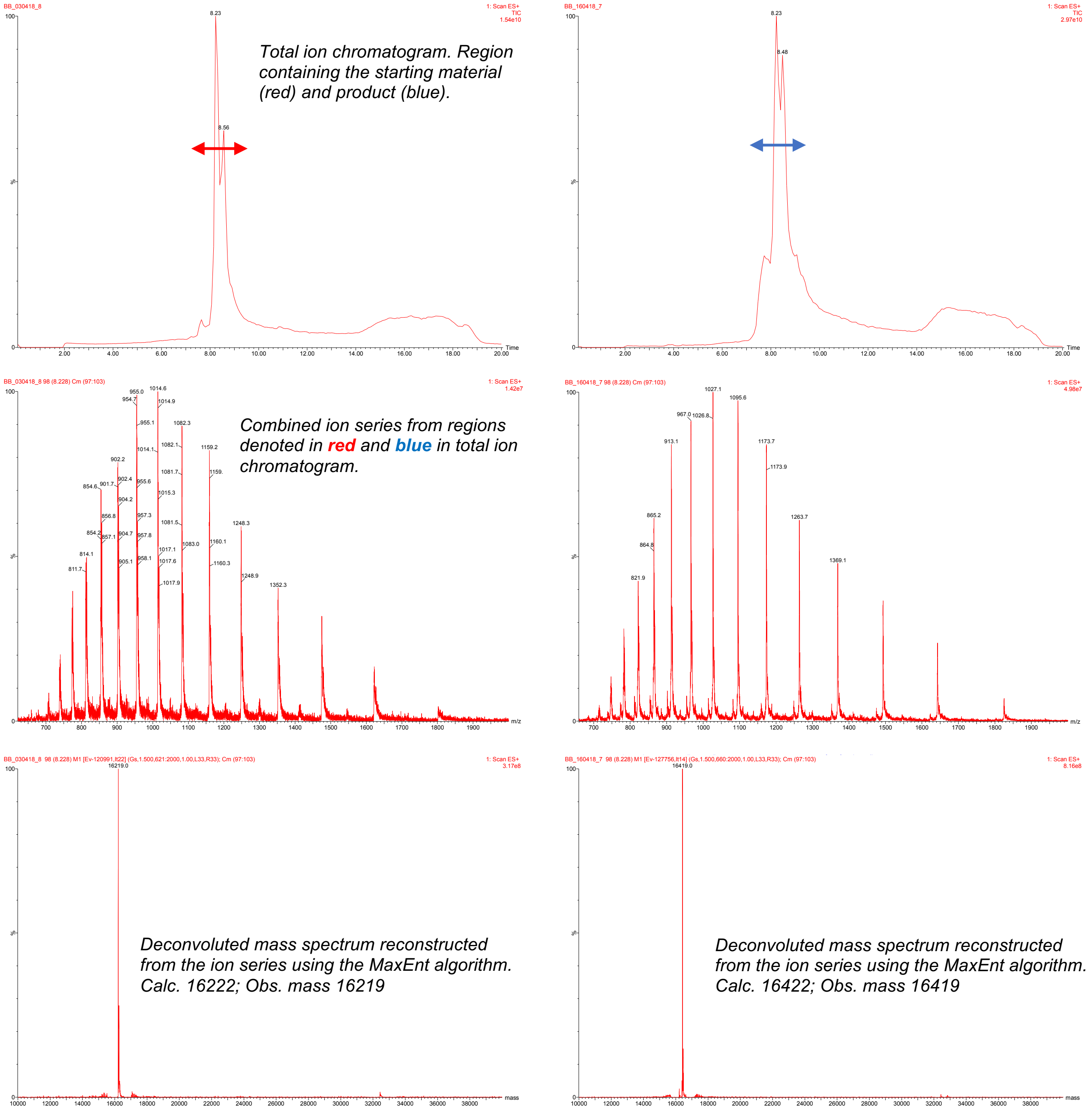

Figure S11. The total ion chromatogram, combined ion series and deconvoluted spectra are shown for the protein starting material and conjugated product.

Veltis HBII

Isotopically Averaged Molecular Weight = 66345 Da.

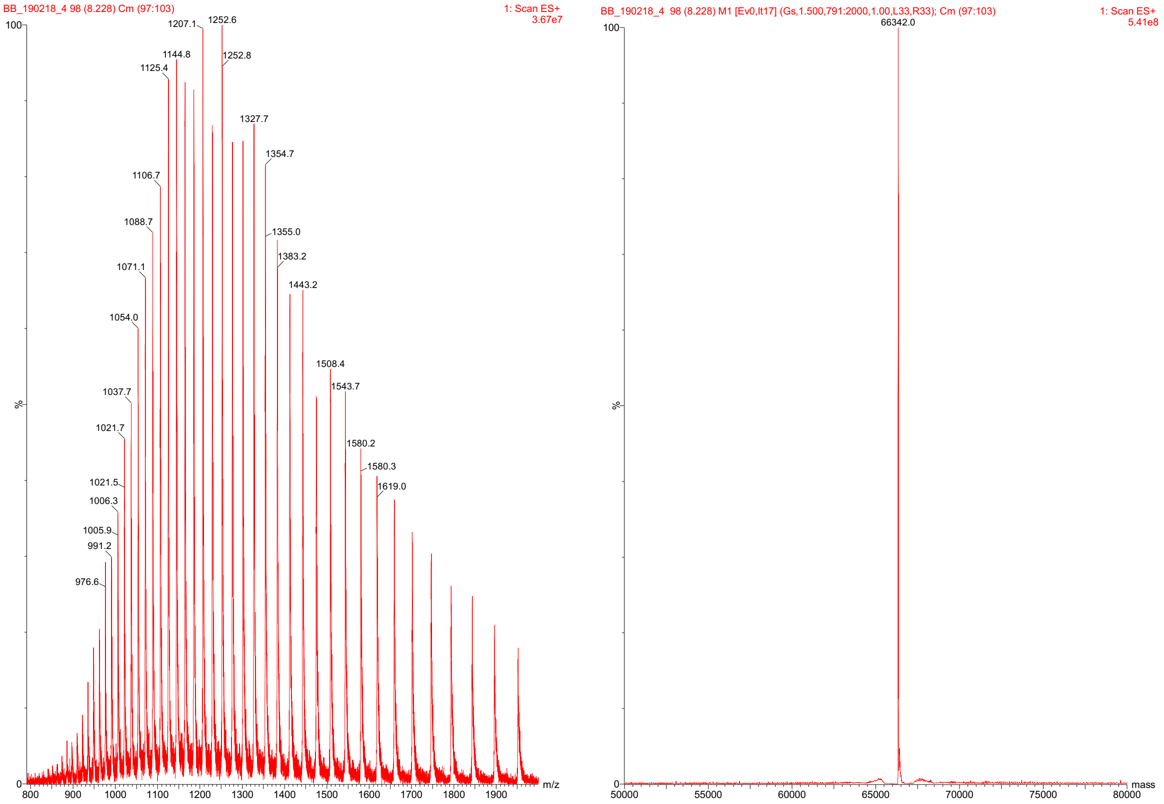

Figure S12. ESI–MS of Veltis HBII.

6. Modification of Veltis HBII

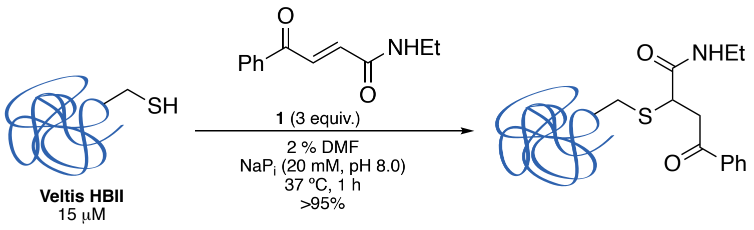

To an eppendorf with 35.2 μL of NaP$_i$ (20 mM, pH 8.0) was added a 3.9 μL aliquot of a stock solution of Veltis HBII (150.7 μM) and the resulting mixture was vortexed for 10 seconds. Afterwards, a 2.17 mM solution of **1** (0.8 μL, 3 equiv.) in DMF was added and the reaction mixed for 1 h at 37 °C. At each reaction time, a 10 μL aliquot was analysed by LC–MS and conversion to the expected product was observed (calculated mass 66533 Da; observed mass, 66532 Da).

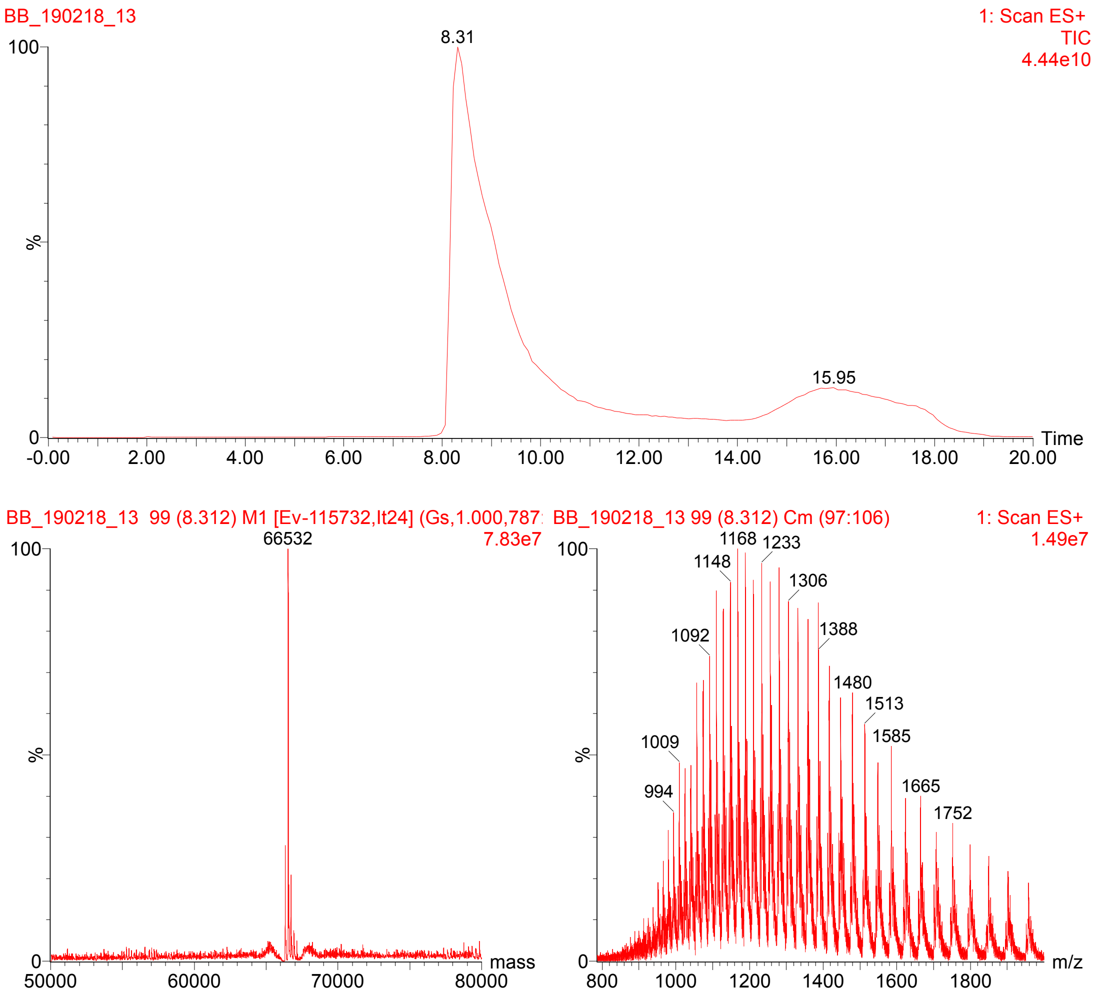

Figure S13. ESI–MS spectra of Veltis HBII-**1** using 3 equiv. after 1 h at 37 °C (NaP$_i$ 20 mM, pH 8).

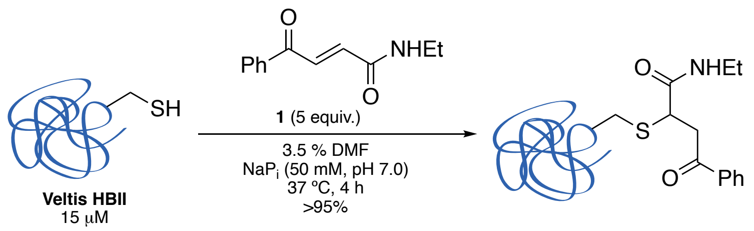

To an eppendorf with 34.6 µL of NaP$_i$ (50 mM, pH 7.0) was added a 3.9 µL aliquot of a stock solution of Veltis HBII (150.7 µM) and the resulting mixture was vortexed for 10 seconds. Afterwards, a 2.17 mM solution of **1** (1.4 µL, 5 equiv.) in DMF was added and the reaction mixed for 4 h at 37 °C. At each reaction time, a 10 µL aliquot was analysed by LC–MS and conversion to the expected product was observed (calculated mass 66533 Da; observed mass, 66521 Da).

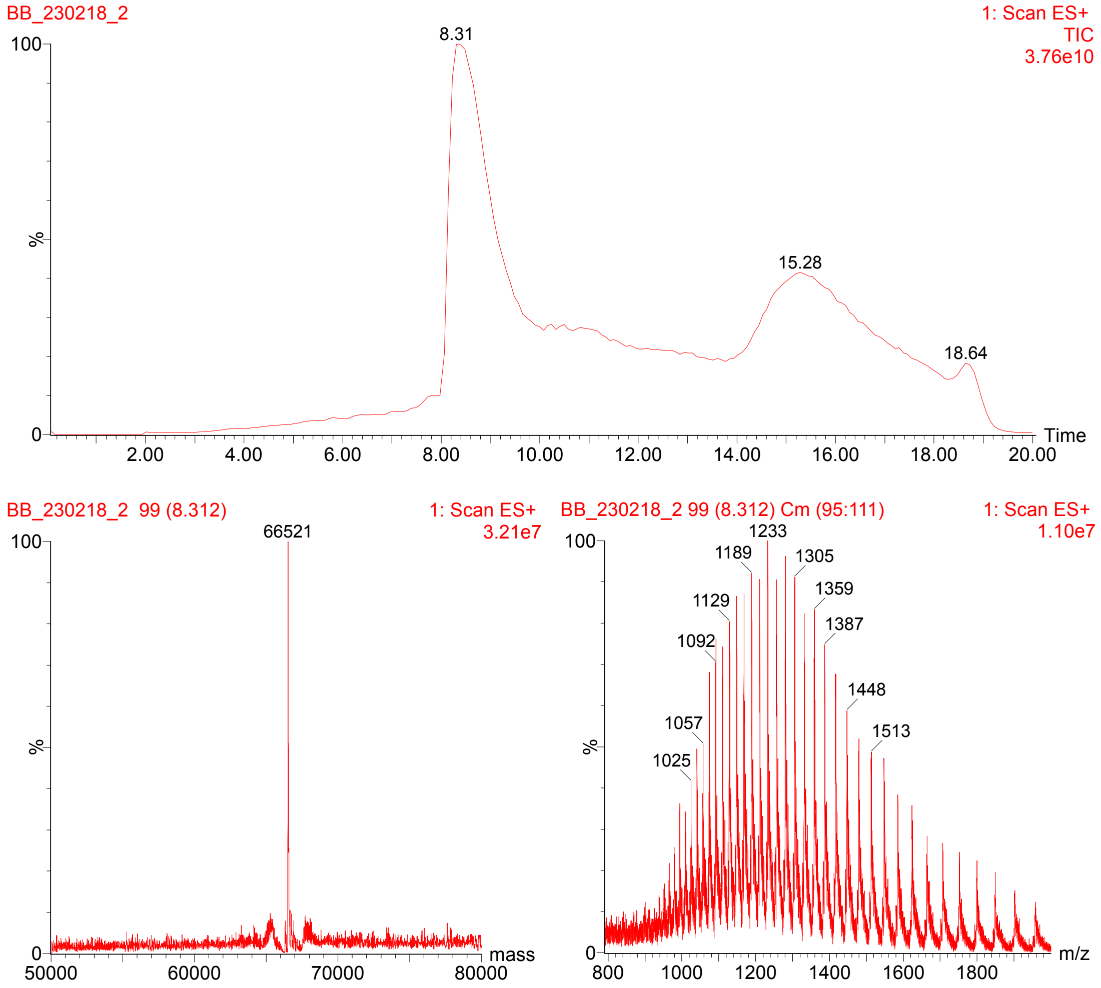

Figure S14. ESI–MS spectra of Veltis HBII-**1** using 5 equiv. after 4 h at 37 °C (NaP$_i$ 50 mM, pH 7).

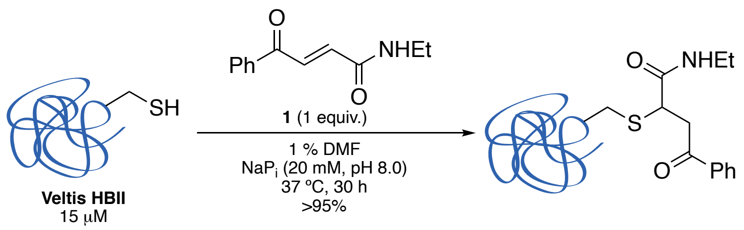

To an eppendorf with 35.7 µL of NaP_i (20 mM, pH 8.0) was added a 3.9 µL aliquot of a stock solution of Veltis V0354 (150.7 µM) and the resulting mixture was vortexed for 10 seconds. Afterwards, a 2.17 mM solution of **1** (0.27 µL, 1 equiv.) in DMF was added and the reaction mixed for 30 h at 37 °C. At each reaction time, a 10 µL aliquot was analysed by LC–MS and conversion to the expected product was observed (calculated mass 66533 Da; observed mass, 66518 Da).

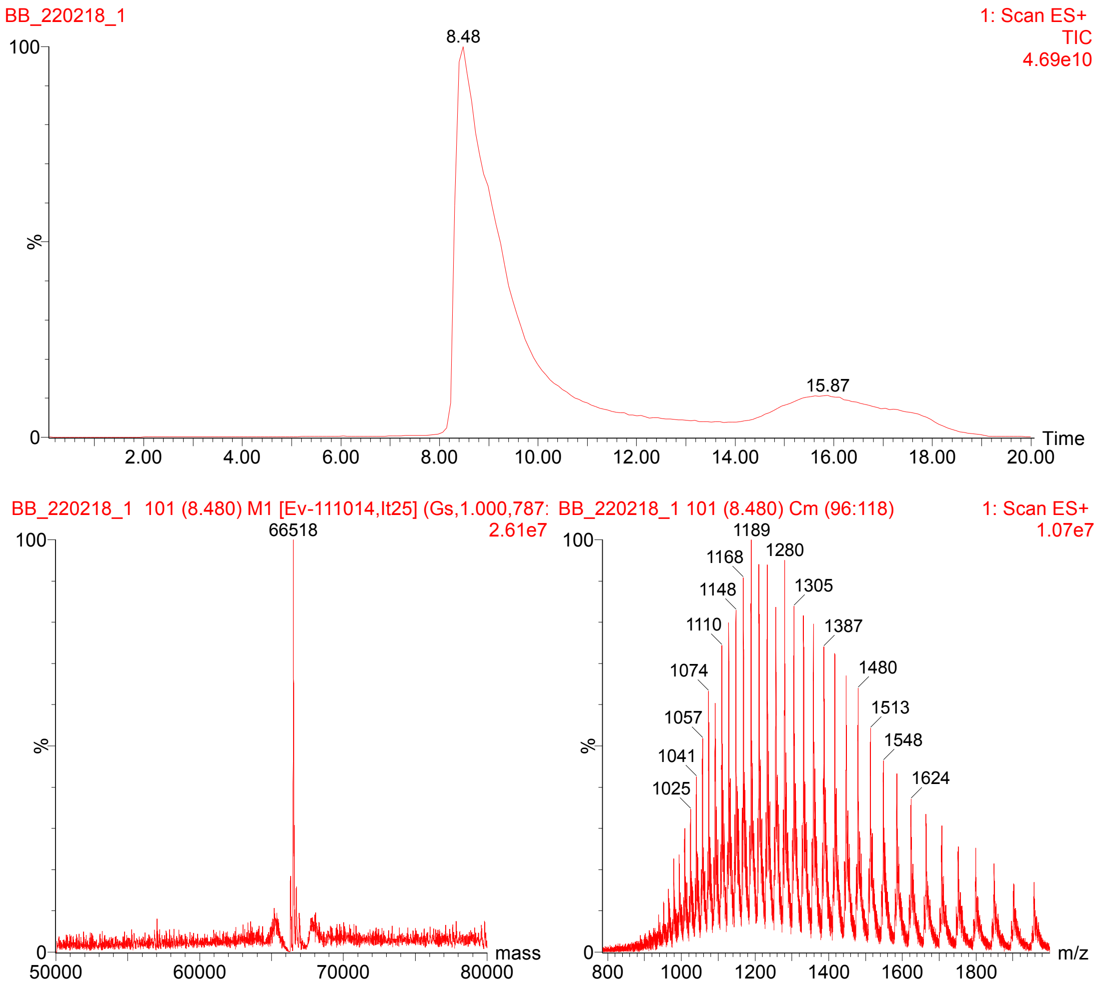

Figure S15. ESI–MS spectra of Veltis HBII-caa using 1 equivalent after 30 h at 37 °C (NaP_i 20 mM, pH 8).

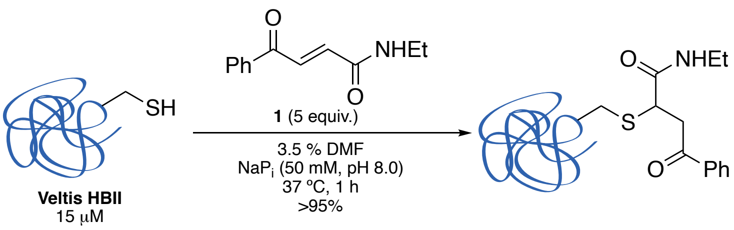

To an eppendorf with 34.6 μL of NaP_i (50 mM, pH 8.0) was added a 3.9 μL aliquot of a stock solution of Veltis V0354 (150.7 μM) and the resulting mixture was vortexed for 10 seconds. Afterwards, a 2.17 mM solution of **1** (1.4 μL, 5 equiv.) in DMF was added and the reaction mixed for 1 h at 37 °C. At each reaction time, a 10 μL aliquot was analysed by LC–MS and conversion to the expected product was observed (calculated mass 66533 Da; observed mass, 66525 Da).

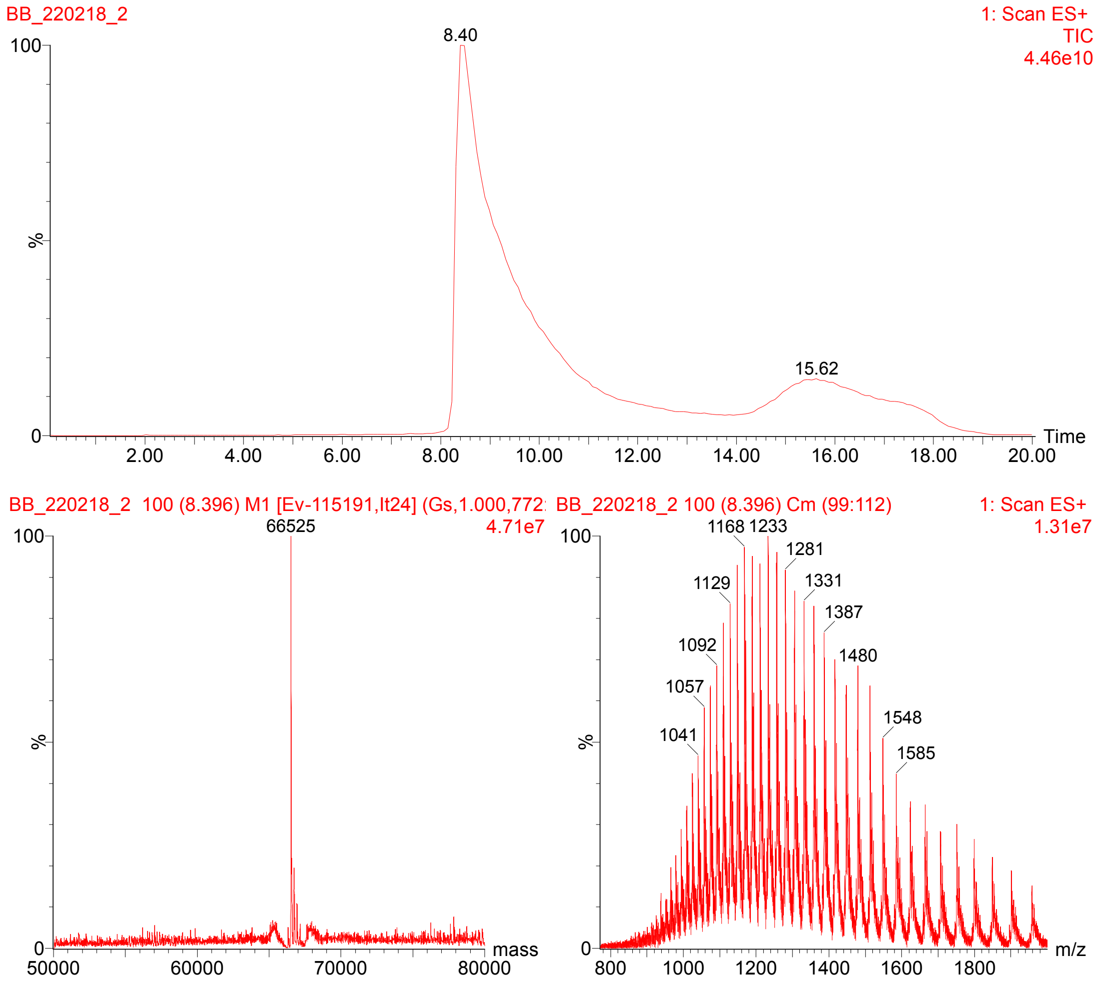

Figure S16. ESI–MS spectra of Veltis HBII-**1** using 5 equiv. after 1 h at 37 °C (NaP_i 50 mM, pH 8)..

Table S1 Optimization studies using Veltis V0354 and **3**.

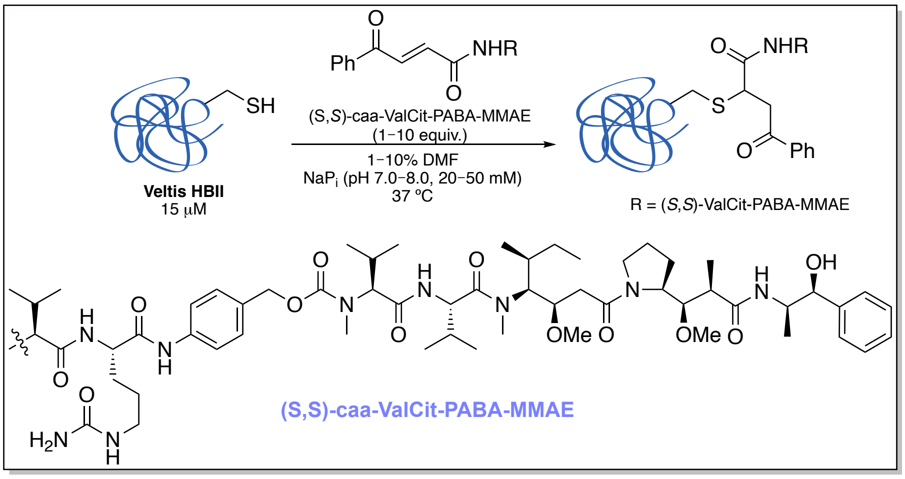

Entry	Equiv. caa-VC-MMAE	Protein (μM)	Buffer (mM)	pH	Time (h)	Conv. (%)	Conv. 2nd mod. (%)
1	1	15	20	7	2	10	0
2	1	15	20	7	17	15	0
3	1	15	20	7	36	20	0
4	5	15	20	7	1	50	40[a]
5	5	15	20	7	3	60	30[a]
6	5	15	20	7	4	65	30[a]
7	1	15	50	8	17	10	0
8	1	15	50	7	36	20	0
9	1.5	15	20	7	2	20	0
10	1.5	15	20	7	24	30	0
11	2	15	20	7	2	30	16
12	2	15	20	7	24	50	0
13	5	15	50	7	24	95	0

[a] full conversion of starting protein, third modification observed

7. Scale-up experiment with caa-(S,S)-VC-MMAE

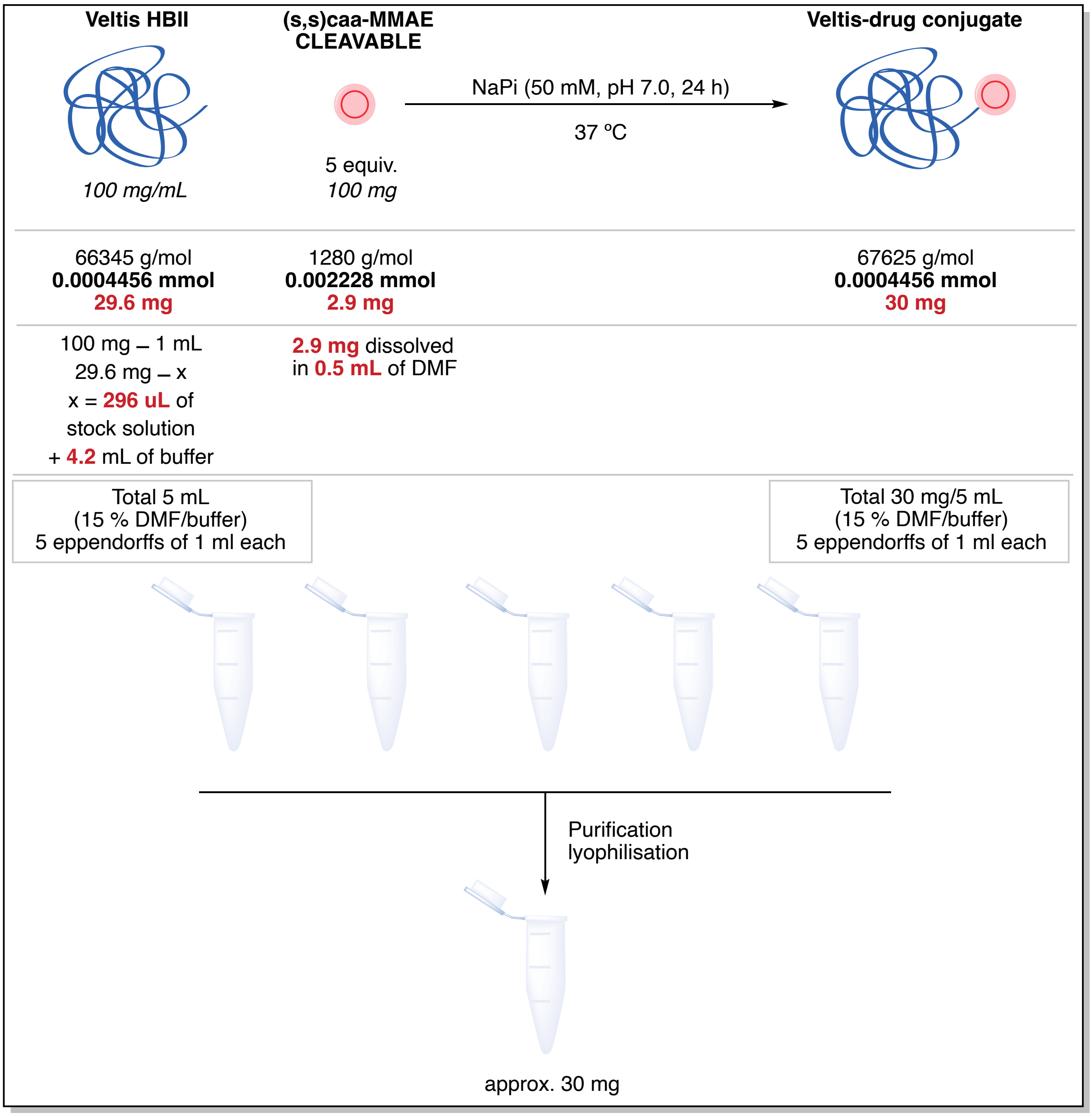

Figure S17. Schematic of bioconjugation scale-up using caa-(S,S)-VC-MMAE.

1) 2.9 mg of caa-(S,S)-VC-MMAE was dissolved in 0.5 mL of DMF and vortexed for 10 s.

2) 296 µL of a 100 mg/mL stock solution of Veltis HBII was dissolved in 4.2 mL of NaP_i (50 mM, pH 7.0) and vortexed for 10 s.

3) In 5 eppendorfs, 0.9 mL of the protein solution was transferred to each of the vial (5 reaction tubes).

4) 0.1 mL of caa-(S,S)-VC-MMAE solution was then added to the each of the 5 reaction tubes and vortexed for 30 seconds.

5) 50 μL of DMF was added to each tube to form a homogeneous solution that was shaken at 37 °C for 24 h.

6) 2 μL of each tube was diluted with 8 μL of NaPi (pH 7.0, 50 mM) in a vial and analysed by LC–MS after 24 h. >90% conversion to the expected product was observed for all the vials (calculated mass 67611 Da; observed mass, 67607 Da).

7) The 5 reaction tubes were combined in 2 15 mL buffer exchange falcons and purified (3 x 30 min).

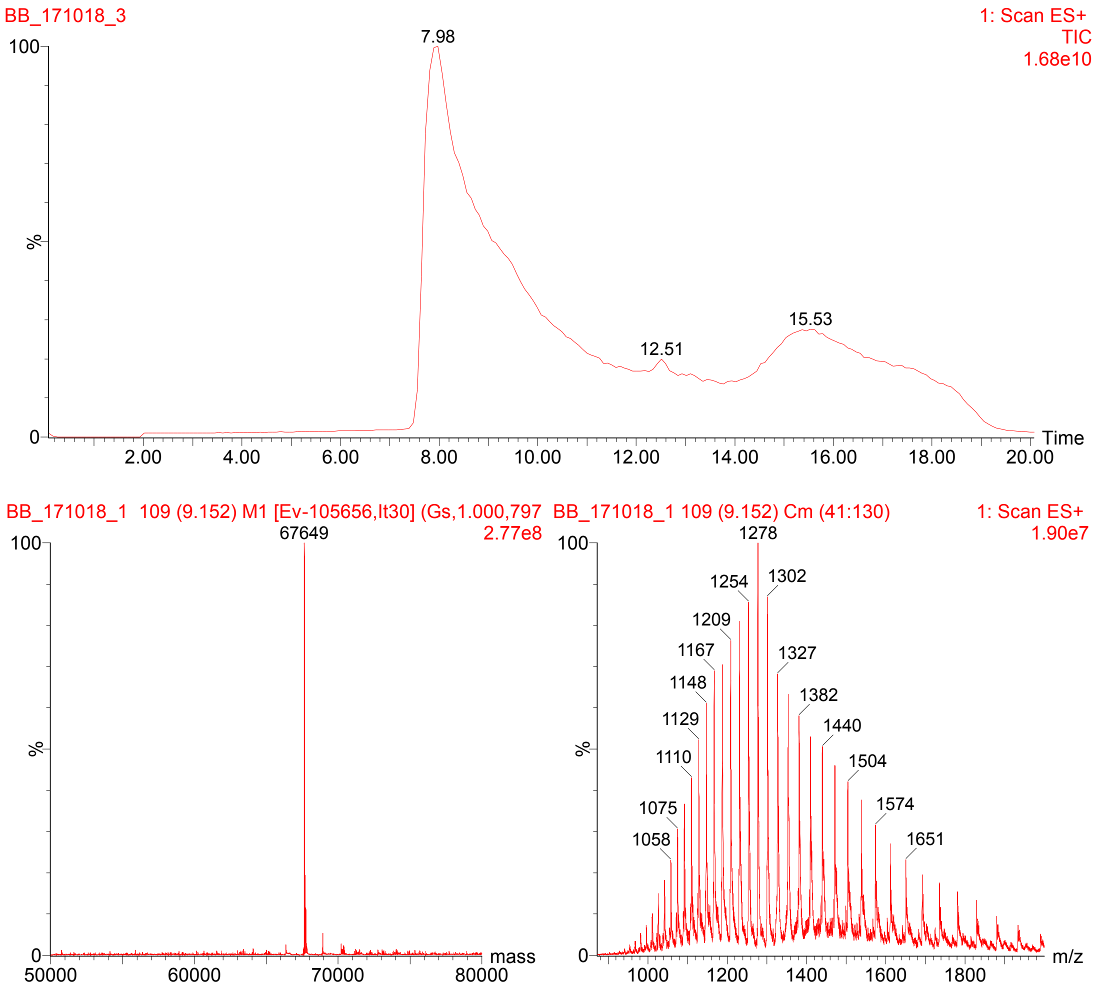

Figure S18. ESI–MS spectra of Veltis HBII-**2** (100 uM), NaPi (50 mM, pH 7.0) after purification.

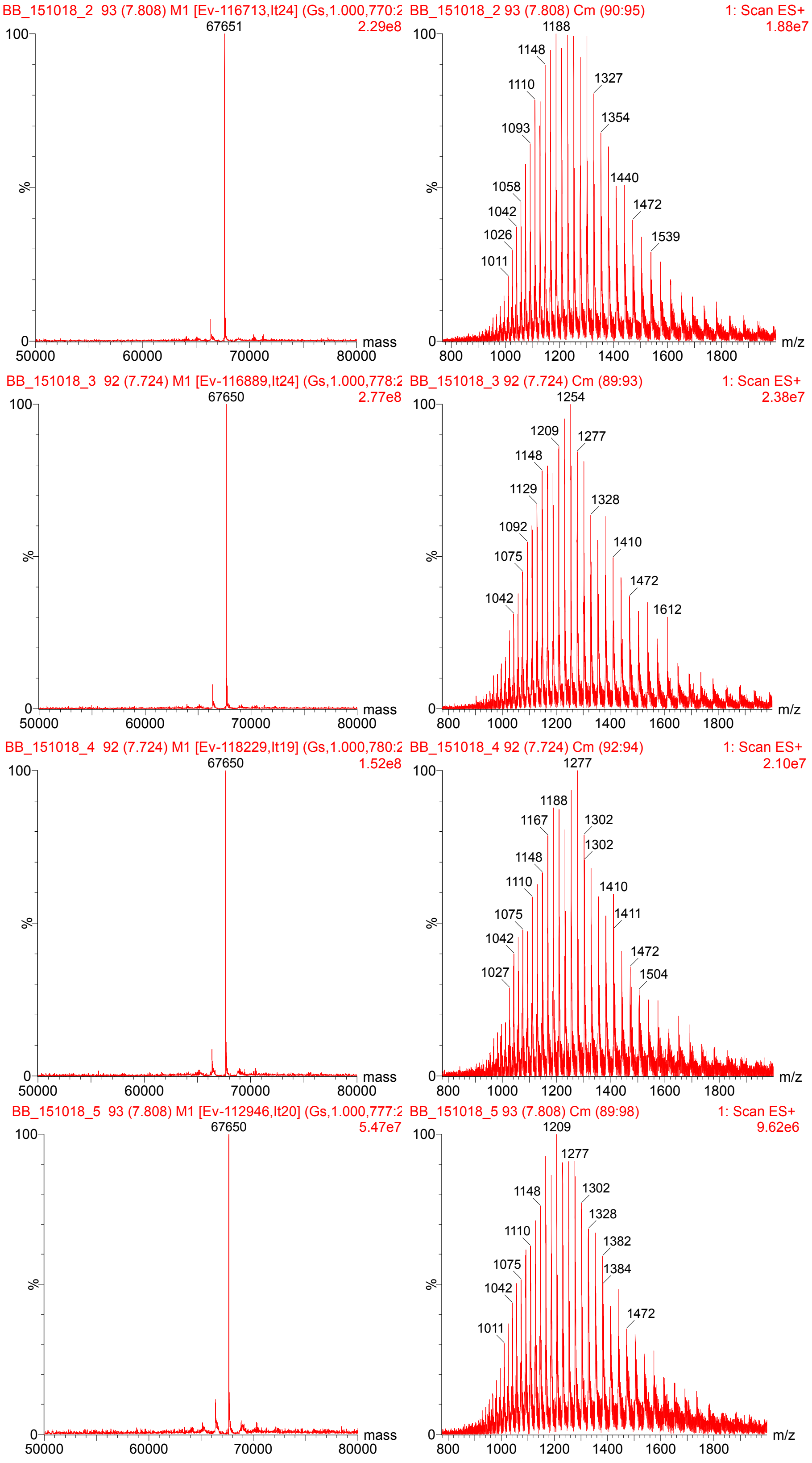
BB_151018_2 93 (7.808) M1 [Ev-116713,It24] (Gs,1.000,770:2
67651
2.29e8
BB_151018_2 93 (7.808) Cm (90:95)
1: Scan ES+
1.88e7
1188
1148
1110
1327
1093
1354
1058
1440
1042
1472
1026
1539
1011
BB_151018_3 92 (7.724) M1 [Ev-116889,It24] (Gs,1.000,778:2
67650
2.77e8
BB_151018_3 92 (7.724) Cm (89:93)
1: Scan ES+
2.38e7
1254
1209
1277
1148
1129
1328
1092
1410
1075
1472
1042
1612
BB_151018_4 92 (7.724) M1 [Ev-118229,It19] (Gs,1.000,780:2
67650
1.52e8
BB_151018_4 92 (7.724) Cm (92:94)
1: Scan ES+
2.10e7
1277
1188
1167
1302
1302
1148
1110
1410
1075
1411
1042
1472
1027
1504
BB_151018_5 93 (7.808) M1 [Ev-112946,It20] (Gs,1.000,777:2
67650
5.47e7
BB_151018_5 93 (7.808) Cm (89:98)
1: Scan ES+
9.62e6
1209
1277
1148
1302
1328
1110
1382
1075
1384
1042
1472
1011
100
%
0
mass
m/z
50000
60000
70000
80000
800
1000
1200
1400
1600
1800

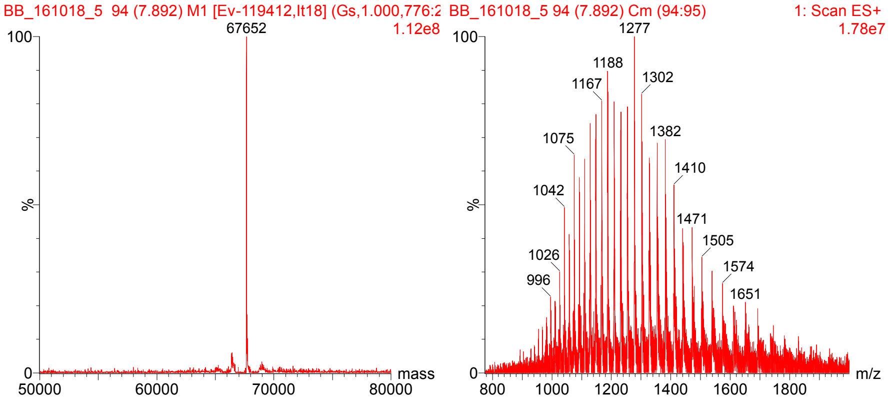

Figure S19. ESI–MS spectra of the 5 reaction vials between Veltis HBII (100 μM) and **2**, NaPi (50 mM, pH 7) after 24 h at 37 ºC.

8. Scale-up experiment with caa-(S,R)-VC-MMAE

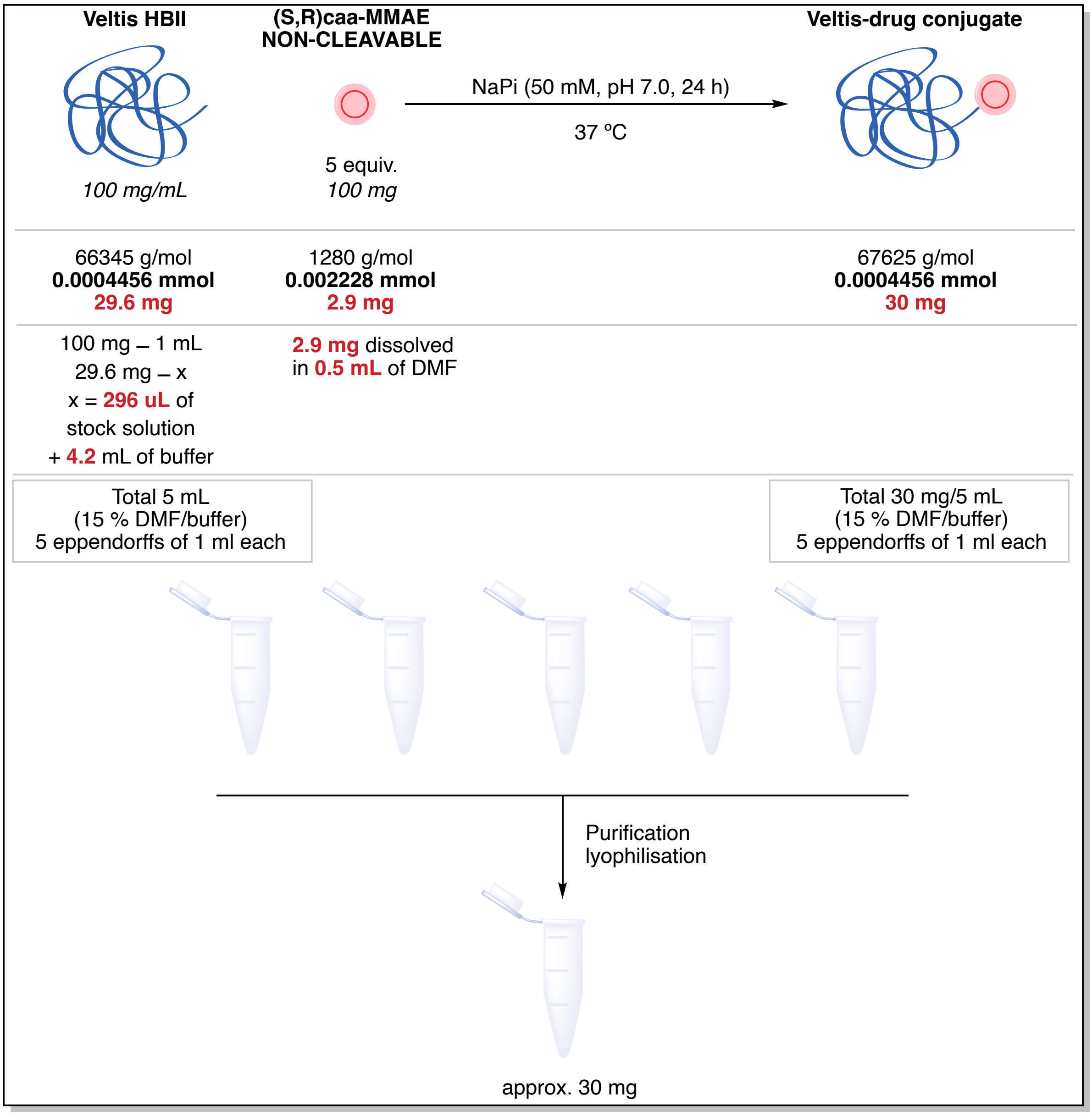

Figure S20. Schematic of the scale-up experiment using caa-(S,R)-VC-MMAE **3**.

1) 2.9 mg of caa-(S,R)-VC-MMAE **3** was dissolved in 0.5 mL of DMF and vortexed.
2) 296 μL of a 100 mg/mL stock solution of Veltis HBII was dissolved in 4.2 mL of NaP_i (50 mM, pH 7.0) and vortexed for 10 s.
3) In 5 eppendorfs, 0.9 mL of the protein solution was transferred to each of the vial (5 reaction tubes).
4) 0.1 mL of **3** solution was then added to the each of the 5 reaction tubes and vortexed for 30 seconds.

5) 50 µL of DMF was added to each tube to form a homogeneous solution that was shaken at 37 °C for 24 h.
6) 2 µL of each tube was diluted with 8 µL of NaPi (pH 7.0, 50 mM) in a vial and analysed by LC–MS after 24 h. >90% conversion to the expected product was observed for all the vials (calculated mass 67611 Da; observed mass, 67607 Da).
7) The 5 reaction tubes were combined in 2 15 mL buffer exchange falcons and purified (3 x 30 min).

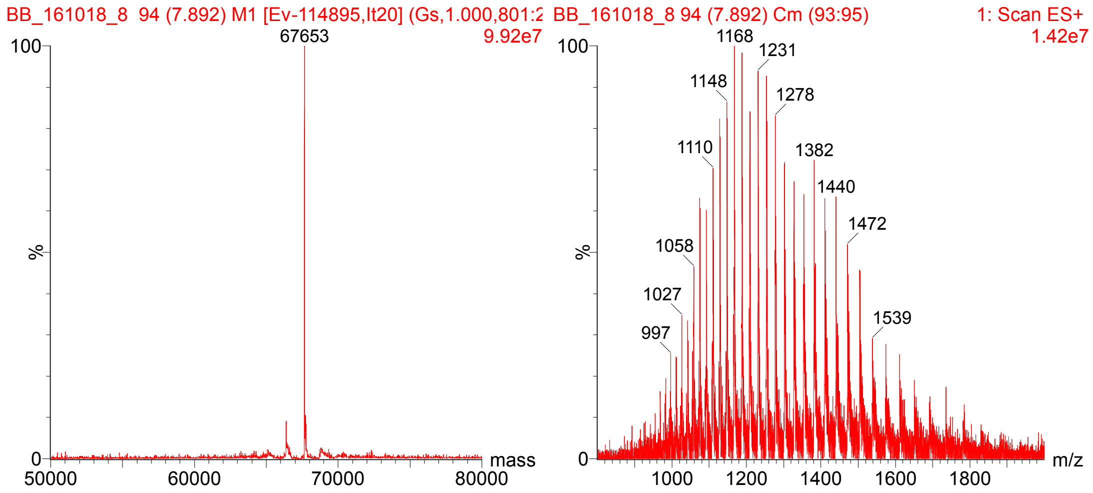

Figure S21. ESI–MS spectra of the reaction between Veltis HBII (100 µM) and **3**, NaPi (50 mM, pH 7) after 24 h at 37 ºC.

9. Optimisation of the reaction with caa-Cy7

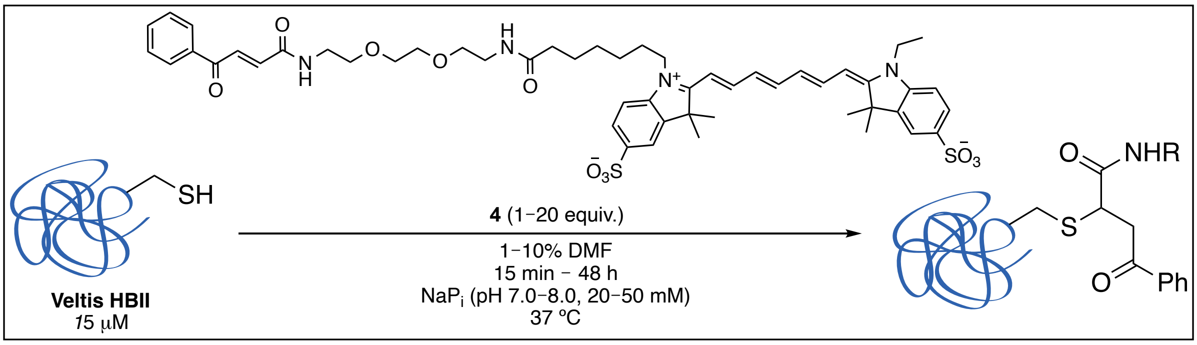

Table S2. Optimisation for the reaction between Veltis HBII and the caa-Cy7 compound.

Entry	Equiv. **4**	Protein (uM)	Buffer (mM)	pH	Time (h)	Conv. (%)	Mod.
1	10	40	50	8	19	70	3
2	10	40	50	7.4	19	75	4
3	100	40	50	8	19	>99	5
4	100	40	50	7.4	19	>99	5
5	1	20	20	8	24	0	0
6	3	20	20	8	24	20	1
7	5	20	50	8	24	60	4
8	5	20	20	7.4	24	60	2
9	5	20	20	7	24	70	1

10. Modification of Veltis HBII with Cy7 fluorophore (small scale)

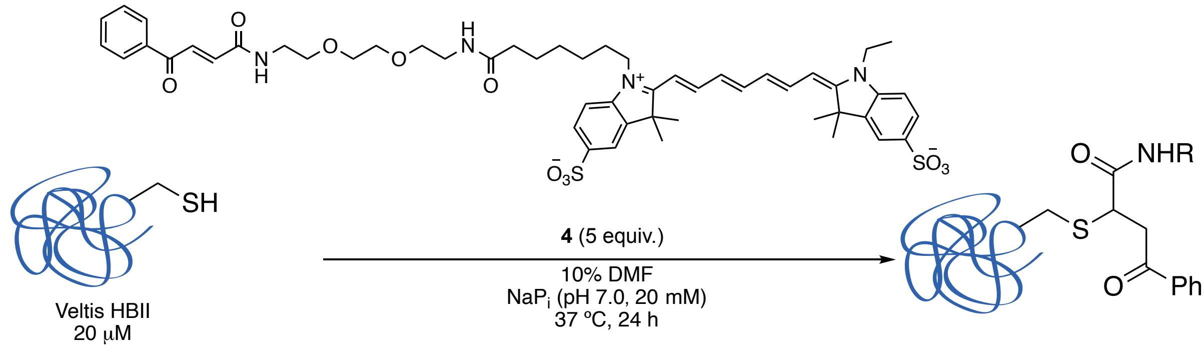

To an eppendorf with 33.3 µL of NaPᵢ (20 mM, pH 7.0) was added a 5.3 µL aliquot of a stock solution of Veltis HBII (150.7 µM) and the resulting mixture was vortexed for 10 seconds. Afterwards, a 10.9 mM solution of caa-Cy7 **4** (0.4 µL, 5 equiv.) in DMF was added and the reaction mixed for 24 h at 37 °C. In the end, a 10 µL aliquot was analysed by LC–MS and 70% conversion to the expected product was observed (calculated mass 67316 Da; observed mass, 67315 Da).

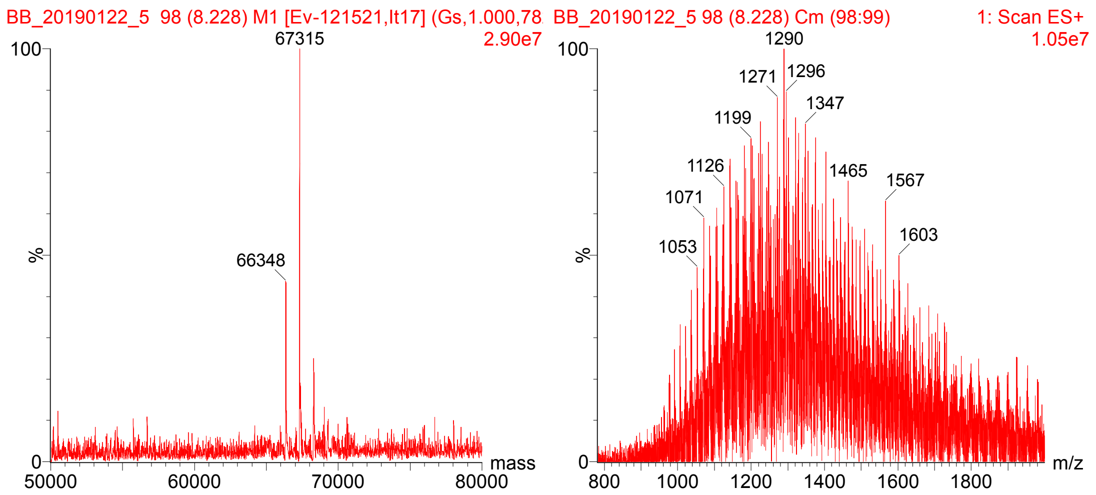

Figure S22. ESI–MS spectra of Veltis HBII with **4**, NaPi (20 mM, pH 7) after 24 h at 37 ºC.

11. Stability in plasma

A 20 μL aliquot of the bioconjugate (10 μM) in NaP_i buffer (50 mM, pH 8.0) was thawed. 1 μL of reconstituted human plasma was added at room temperature and the resulting mixture vortexed for 10 seconds. The resulting reaction mixture was then mixed at 37 ºC. After 24 h aliquot of the reaction mixture was analysed by LC–MS. No significant degradation of the adduct was observed.

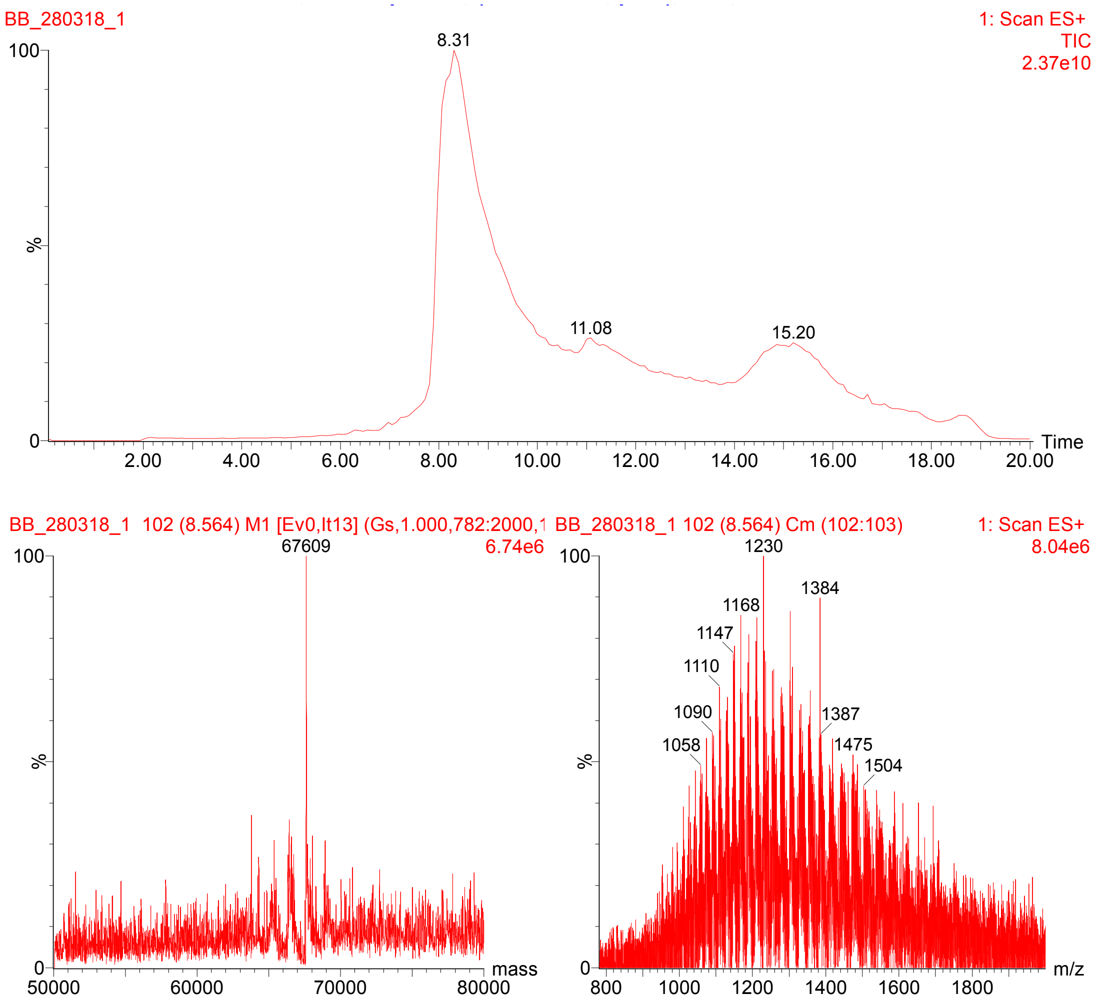

Figure S23. ESI–MS spectra of Veltis HBII-**2** in the presence of plasma after 24 h at 37 ºC.

12. Circular dichroism analysis of Veltis HBII

Circular dichroism (CD) spectroscopy was used to analyse protein secondary structure in solution. Samples were concentrated to 10 nM in NaPi buffer (50 mM, pH 8.0). CD measurements were recorded using a Chirascan spectrophotometer equipped with a Quantum TC125 temperature control unit (25 °C). The data was acquired in a 0.1 cm path length with a response time of 1 s, a per-point acquisition delay of 5 ms and a pre- and post-scan delay of 50 ms. Spectra were averaged over three scans, in a wavelength range from 200 nm to 260 nm, and the spectrum from a blank sample containing only buffer was subtracted from the averaged data.

13. SDS-PAGE analysis

The reaction solution (5.0 mL) was transferred to a tube, and NuPAGE LDS Sample Buffer (4x, 2.5 mL), NuPAGE Reducing Agent (10x, 1 mL), and H_2O (1.5 mL) were added to the tube. The solution was heated at 70 °C for 10 min. The heated solution was loaded to NuPAGE Bis-Tris mini gel (10x 10 cm) with 4–12% gradient polyacrylamide concentration, and then the conjugation reaction was analysed by electrophoresis (200 V). The buffering system employed was 1x SDS Running Buffer (NuPAGE MES SDS Running Buffer, 20x, pH 7.3, 50 to 950 mL deionised water). 500 mL of NuPAGE antioxidant was added to each 200 mL 1x SDS running buffer. After 35 min, the intensities of fluorescence were analysed. Then, the gel was stained with 0.5% of Ruby. The gel shook gently overnight at room temperature and read the day after. After wash the gel, coomassie (0.5%) was added and the gel was read 2 h after shaking at room temperature.

14. Measuring hydrodynamic radiuses and binding curves

The hydrodynamic radius of Alexa-647 labelled FcRn alone and with Albumin and Albumin-drug-conjugate were measured with Fluidity one W (Fluidic analytics) that is based on microfluidic diffusional sizing.[1] In microfluidic diffusional sizing, 6 µl of the sample, containing 42 nM of FcRn and desired amount of Albumin in a buffer (50 mM NaP_i, 150 mM NaCl, pH 5) was used for each measurement point. All samples were incubated at least for 1 h in 25 ºC prior to measurement. The FcRn-Albumin interaction was assessed with an increase of hydrodynamic radius of FcRn with the increasing Albumin concentration. The increase of FcRn size is due to interaction with Albumin. Since, only FcRn is labelled, it appears larger when bound to another protein than when free in a solution.

Determination of Binding Constants

Non-cooperative binding model was used to obtain the dissociation constant K_D by fitting the change in hydrodynamic radius upon FcRn binding to Albumin:

$$\boldsymbol{r} = \left(\left(\frac{[\boldsymbol{rVeltis}]+\boldsymbol{n}\cdot[\boldsymbol{FcRn}]+\boldsymbol{K_D}}{2} - \sqrt{\left(\frac{[\boldsymbol{rVeltis}]+\boldsymbol{n}\cdot[\boldsymbol{FcRn}]+\boldsymbol{K_D}}{2}\right)^2 - [\boldsymbol{rVeltis}]\cdot\boldsymbol{n}\cdot[\boldsymbol{FcRn}]}\right)\frac{\Delta \boldsymbol{r_{s,tot}}}{\boldsymbol{n}\cdot[\boldsymbol{FcRn}]}\right) + \boldsymbol{r_0} \qquad \textbf{(x)}$$

Where [Albumin] is the concentration of Albumin in the solution that was varied and is x-axis in the binding curve. r is the measured size of FcRn in giving [Albumin] and [FcRn] is concentrations of protein in solution. K_D is dissociation constant (in M), n is a number of binding sites (here n=1), $\Delta r_{s,tot}$ is the difference between the radius of unbound FcRn and radius of FcRn-Albumin complex, r_0 is the radius of unbound protein.[2]

15. Development of ovarian cancer mice model and albumin-conjugates administration

Subcutaneous tumours were induced in female Athymic Nude mice (from Charles River Laboratories International, 6 weeks, n=5) by injection of 1.5×10^6 SK-OV-3 ovarian cancer cells (ATCC), suspended in 50 µL of sterile 1x PBS solution and 50 µL of Matrigel® Matrix (Corning). For determination of tumour growth, individual tumours were measured (2-3 times per week) using calliper and tumour volume was calculated by: Tumour volume (mm^3) = width × ($length^2$) / 2. Treatments began when tumour volume reached about 100 mm^3, by injecting intravenously (tail-vein injection) 2 mg/Kg of each compound during 7 consecutive days. Measurement of animal weight was performed 3 times per week. At the end of the trial, all tumours were weighed and collected along with major organs (kidneys, spleen, liver, lungs, heart, and intestines) for histology and pathology analysis. All experimental protocols were approved by the iMM Animal Care and Use Committee and were in compliance with European guidelines for animal use.

16. Biodistribution analysis of albumin-conjugates

Sub-cutaneous tumours were induced in female Athymic Nude mice (from Charles River Laboratories International, 6 weeks, n=6) by injection of 1.5×10^6 SK-OV-3 ovarian cancer cells (ATCC), suspended in 50 µL of sterile 1x PBS solution and 50 µL of Matrigel® Matrix (Corning).

Non-invasive longitudinal monitoring of biodistribution of albumin-conjugates in major organs and tumour was followed by scanning mice with the IVIS Spectrum-bioluminescent and fluorescent imaging system (Lumina from Perkin Elmer, Calliper Life Sciences) from mice bearing ovarian tumours from SK-OV-3 cells (n=6). Whole-animal imaging was performed at the indicated time points (0, 1, 3, 6, 24 and 48 hours). After 48 hours, mice were sacrificed, and the organs harvested and imaged with the IVIS Spectrum-bioluminescent and fluorescent imaging system.

17. Statistics

We performed all statistical analyses with two-tailed Student's t-test unless noted otherwise. Results are represented as mean±s.e.m. unless noted otherwise. No

animal or sample was excluded from the analysis. The P values are *P≤0.01, **P≤0.001 and ***P≤0.0001.

18. Cathepsin B Assay using caa-(S,S)-VC-MMAE and caa-(S,R)-VC-MMAE

Compounds **2** and **3** were dissolved in biological grade DMF to a concentration of 10 mM. These were then diluted into MilliQ water to a concentration of 100 μM. Next the assay buffer was prepared (65 mM MES, 5 mM EDTA, 2 mM DTT, pH 6.5) and filtered. A fresh stock of DTT (200 mM) in MilliQ water was also prepared for enzyme activation. Cathepsin B (Merck 219362-50ug) was prepared in aliquots at 250 μg/mL. For a reaction, 30 μL of 100 μM compound **2** or **3** stock was combined with 60 μL of assay buffer and 5 μL of 200 mM DTT. 5 μL of Cathepsin B enzyme was then added, and the reaction incubated with shaking at 37°C for 12 hr. 15 μL aliquots were removed at t = 0 h, 3h, and 12 h and flash frozen for further analysis via LCMS. For the control reaction, and extra 5 μL of assay buffer was added in place of the cathepsin B enzyme. The reaction was performed in duplicate with the recorded ESI-MS ES+ spectra averaged. UV chromatograms are not shown as the compound concentration was too low for sufficient absorbance detection by the LCMS instrument used.

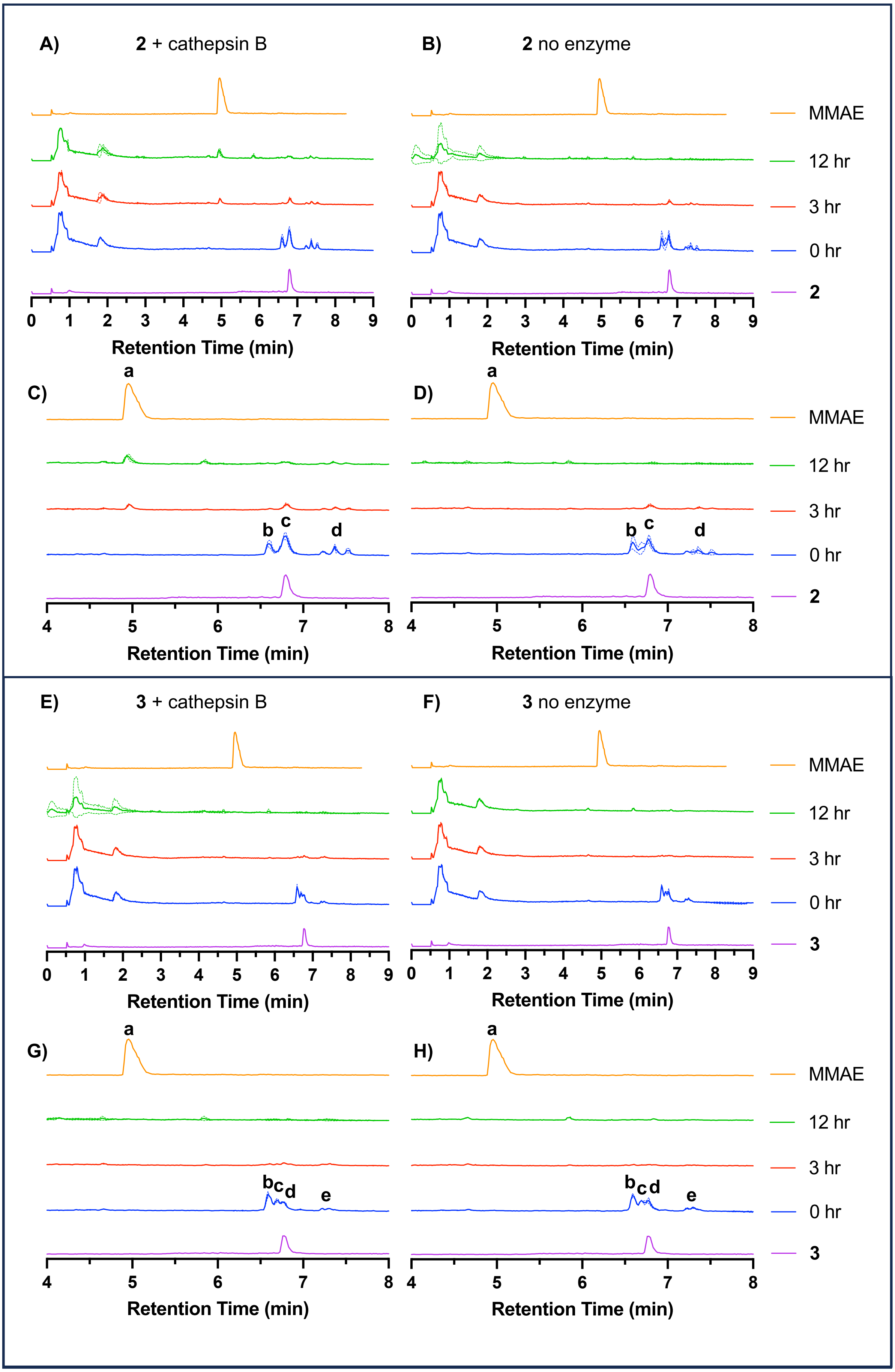

A)
2 + cathepsin B
B)
2 no enzyme
MMAE
12 hr
3 hr
0 hr
2
0 1 2 3 4 5 6 7 8 9
Retention Time (min)
C)
D)
a
b c d
4 5 6 7 8
Retention Time (min)
E)
3 + cathepsin B
F)
3 no enzyme
3
G)
H)
bcd
e

Figure S24. ESI-MS positive mode spectra of **2** and **3** after 0 h, 3 h, and 12 h incubation at 37°C with or without cathepsin B enzyme. Spectra are the average of two duplicates, with the dotted line representing ± s.e.m. Reference spectra for the starting compounds **2** and **3** as well as MMAE are included. **A) 2** with cathepsin B, **B) 2** without cathepsin B added, **C)** zoom of **2** with cathepsin B, **D)** zoom of **2** without cathepsin B added. **E) 3** with cathepsin B, **F) 3** without cathepsin B added, **G)** zoom of **3** with cathepsin B, **H)** zoom of **3** without cathepsin B added.

MMAE release was only detected in the presence of cathepsin B enzyme for compound **2**. No MMAE was detected in the control assays without enzyme or for compound **3** in the presence of cathepsin B. This supports the stability of both linker constructs towards hydrolysis as well as the stability of caa-(S,R)-VC-MMAE against protease cleavage. The DTT adducts for compounds **2** and **3** as well as a **2-DTT-2** and **3-DTT-3** dimer were also detected. As DTT was needed for enzyme activation and both compounds **2** and **3** contained caa functionalities, these adducts were unavoidable. The low solubility of both linkers likely contributed to their diminished signals over time.

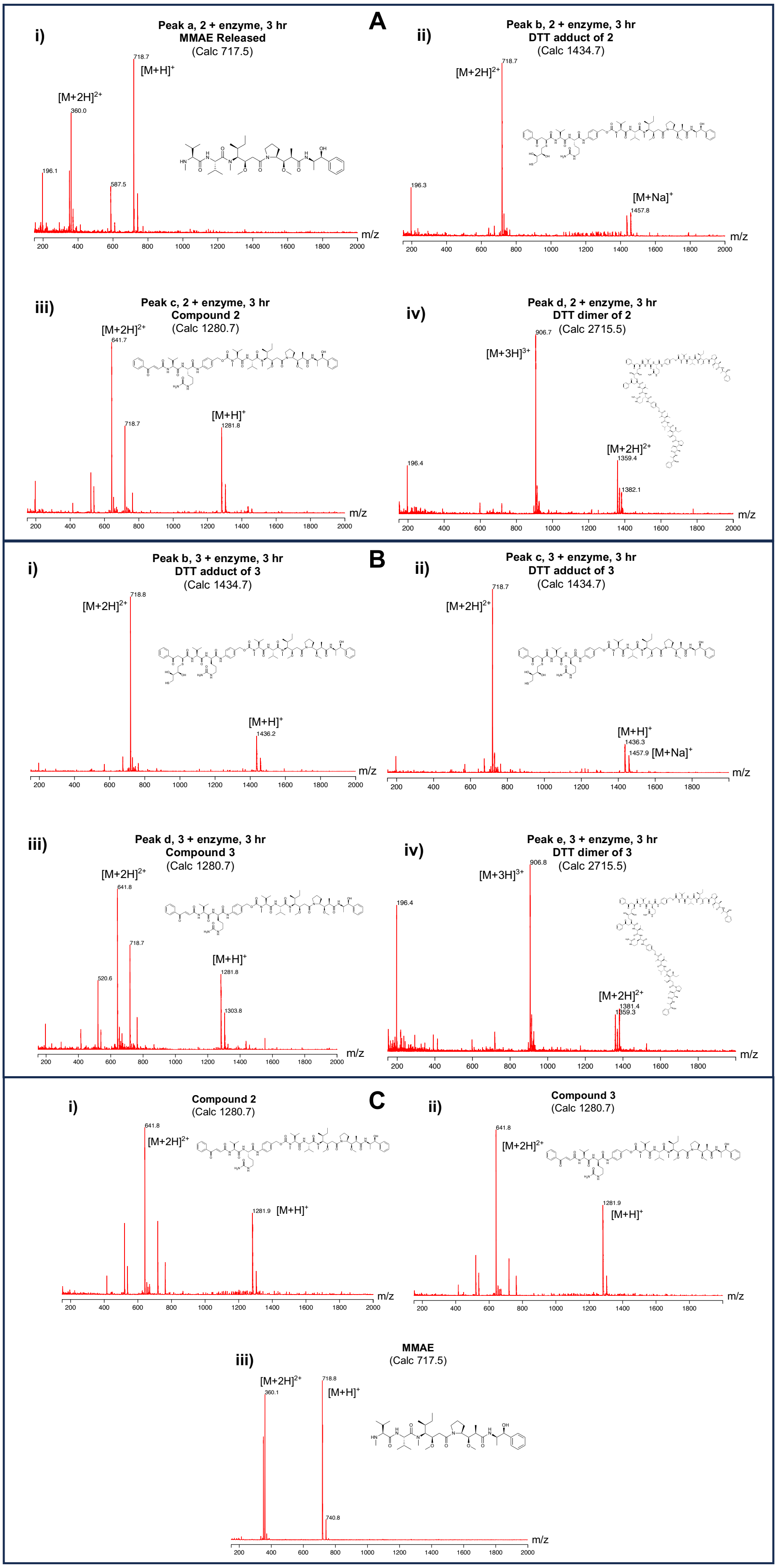
A
i)
Peak a, 2 + enzyme, 3 hr
MMAE Released
(Calc 717.5)
[M+H]+
[M+2H]2+
ii)
Peak b, 2 + enzyme, 3 hr
DTT adduct of 2
(Calc 1434.7)
[M+2H]2+
[M+Na]+
iii)
Peak c, 2 + enzyme, 3 hr
Compound 2
(Calc 1280.7)
[M+2H]2+
[M+H]+
iv)
Peak d, 2 + enzyme, 3 hr
DTT dimer of 2
(Calc 2715.5)
[M+3H]3+
[M+2H]2+
B
i)
Peak b, 3 + enzyme, 3 hr
DTT adduct of 3
(Calc 1434.7)
[M+2H]2+
[M+H]+
ii)
Peak c, 3 + enzyme, 3 hr
DTT adduct of 3
(Calc 1434.7)
[M+2H]2+
[M+H]+
[M+Na]+
iii)
Peak d, 3 + enzyme, 3 hr
Compound 3
(Calc 1280.7)
[M+2H]2+
[M+H]+
iv)
Peak e, 3 + enzyme, 3 hr
DTT dimer of 3
(Calc 2715.5)
[M+3H]3+
[M+2H]2+
C
i)
Compound 2
(Calc 1280.7)
[M+2H]2+
[M+H]+
ii)
Compound 3
(Calc 1280.7)
[M+2H]2+
[M+H]+
iii)
MMAE
(Calc 717.5)
[M+2H]2+
[M+H]+
m/z

Figure S25. Peak identification by m/z for representative ESI-MS traces. **A)** M/z for peaks in **2** with cathepsin B, 3 hr. **B)** M/z for peaks in **3** with cathepsin B, 3 hr. **C)** m/z for reference standards of **2**, **3**, and MMAE. Peak letter labels correspond to those in Figure S24. Major ions are labelled and compound structures are shown.

19. General LC-MS Conditions

All LC-MS studies on proteins and peptides were carried out using a Waters SQD2 mass spectrometer coupled to an Acquity UPLC system. Proteins were analysed with an Acquity UPLC BEH300 C4 column (1.7μm) and peptides were analysed with an Acquity UPLC BEH C18 column (1.7 μm). Solvent A (99.9% MilliQ water and 0.1% formic acid) and solvent B (99.9% LCMS-grade acetonitrile and 0.1% formic acid) were used for both methods with a total flow rate of 0.2 mL/min. For peptide analysis, the following gradient was used: 5% B for 0.5 minutes, then 5% B to 90% B in 5.5 minutes, then 90% B for 2.5 minutes, then 90% B to 5% B in 0.25 minutes, then 5% B for 0.5 minutes. For protein analysis, the following gradient was used: 5%B for 2 minutes 5% B to 72% B in 15 minutes, then 72% B for 1.5 minutes, then 72% B to 5% B in 0.25 minutes, then 5% B for 1.25 minutes. The capillary voltage of the electrospray source was 3.0kV and the cone voltage was 30 V. Nitrogen was the desolvation gas at a flow rate of 800 L/h. Ion series were deconvoluted with the MaxEnt1 function on MassLynx software (v. 4.1)

20. References

(1) Yates, E. V.; Müller, T.; Rajah, L.; De Genst, E. J.; Arosio, P.; Linse, S.; Vendruscolo, M.; Dobson, C. M.; Knowles, T. P. Latent analysis of unmodified biomolecules and their complexes in solution with attomole detection sensitivity. *Nat. Chem.* **2015**, *7* (10), 802–809.

(2) Wilkinson, K. D. Quantitative Analysis of Protein-Protein Interactions. In *Protein-Protein Interactions: Methods and Applications*, Fu, H. Ed.; Humana Press, 2004; pp 15-31.
